# Supplementary material for: Association between composite dietary antioxidant indices and anemia: NHANES 2003–2018
Source: PLoS One. 2025 Jan 2;20(1):e0316397. doi: 10.1371/journal.pone.0316397 (PMC11694997; doi:10.1371/journal.pone.0316397)
Supplement: S1 Table — (DOCX) [file pone.0316397.s003.docx]

**Supplementary Table 1.** The weighted logistic regression analysis of the association between CDAI and HB.

| **Characteristics** | **Univariable analysis** | | **Multivariable analysis** | |
| --- | --- | --- | --- | --- |
|  | **OR (95% CI)** | ***P* value** | **OR (95% CI)** | ***P* value** |
| **CDAI** | 0.94 (0.93,0.96) | **<0.001** | 0.97 (0.95,0.98) | **<0.001** |
| **Age (years)** |  |  |  |  |
| **≤40** | ref |  | ref |  |
| **>40** | 0.76 (0.69,0.84) | **<0.001** | 0.73 (0.65,0.82) | **<0.001** |
| **Sex** |  |  |  |  |
| Female | ref |  |  |  |
| Male | 0.40 (0.36,0.45) | **<0.001** | 0.44 (0.39,0.49) | **<0.001** |
| **Race** |  |  |  |  |
| Non-Hispanic black | ref |  | ref |  |
| Non-Hispanic white | 0.21 (0.19,0.24) | **<0.001** | 0.21 (0.19,0.24) | **<0.001** |
| Mexican American | 0.32 (0.28,0.37) | **<0.001** | 0.31 (0.27,0.36) | **<0.001** |
| Other Hispanic | 0.33 (0.29,0.38) | **<0.001** | 0.33 (0.29,0.38) | **<0.001** |
| **Marital status** |  |  |  |  |
| Divorced | ref |  |  |  |
| Living with partner | 0.83 (0.64,1.09) | 0.181 |  |  |
| Married | 0.85 (0.72,1.02) | 0.082 |  |  |
| Never married | 0.90 (0.74,1.10) | 0.298 |  |  |
| Separated/Windowed | 1.47 (1.11,1.94) | **0.008** |  |  |
| **Education levels** |  |  |  |  |
| High school | ref |  | ref |  |
| Less than high school | 1.22 (1.02,1.45) | **0.032** | 0.95 (0.67,1.35) | 0.780 |
| More than high school | 0.70 (0.61,0.80) | **<0.001** | 1.67 (1.29,2.16) | **<0.001** |
| **BMI** |  |  |  |  |
| Normal | ref |  |  |  |
| Thin | 0.87 (0.60,1.26) | 0.453 |  |  |
| Obese | 1.11 (0.99,1.24) | 0.077 |  |  |
| Overweight | 0.82 (0.72,0.93) | **0.002** |  |  |
| **PIR** |  |  |  |  |
| ≥2.14 | ref |  | ref |  |
| <2.14 | 1.77 (1.59,1.97) | **<0.001** | 1.30 (1.16,1.46) | **<0.001** |
| **Smoke** |  |  |  |  |
| No | ref |  | ref | ref |
| Yes | 0.50 (0.44,0.56) | **<0.001** | 1.37 (1.24,1.52) | **<0.001** |
| **Drink** |  |  |  |  |
| No | ref |  |  |  |
| Yes | 0.71 (0.65,0.78) | **<0.001** | 0.78 (0.70,0.86) | **<0.001** |
| **Hypertension** |  |  |  |  |
| No | ref |  | ref |  |
| Yes | 1.66 (1.52,1.82) | **<0.001** | 1.37 (1.24,1.52) | **<0.001** |
| **Hyperlipidemia** |  |  |  |  |
| No | ref |  |  |  |
| Yes | 0.97 (0.90,1.08) | 0.765 |  |  |
| **Diabetes** |  |  |  |  |
| Borderline | ref |  |  |  |
| No | 0.59 (0.43,0.80) | **<0.001** |  |  |
| Yes | 1.47 (1.06,2.04) | **0.022** |  |  |
| **Cancer** |  |  |  |  |
| No | ref |  |  |  |
| Yes | 1.45 (1.25,1.68) | **<0.001** | 1.45 (1.22,1.72) | **<0.001** |
