# Supplementary material for: Association between composite dietary antioxidant indices and anemia: NHANES 2003–2018
Source: PLoS One. 2025 Jan 2;20(1):e0316397. doi: 10.1371/journal.pone.0316397 (PMC11694997; doi:10.1371/journal.pone.0316397)
Supplement: Attachment — Submitted filename: PONE-D-24-28259_R1_reviewer.pdf [file pone.0316397.s006.pdf]

PLOS ONE

Association between composite dietary antioxidant indices and anemia: NHANES 2003–2018

--Manuscript Draft--

**Manuscript Number:**	PONE-D-24-28259R1
**Article Type:**	Research Article
**Full Title:**	Association between composite dietary antioxidant indices and anemia: NHANES 2003–2018
**Short Title:**	CDAI and anemia
**Corresponding Author:**	Qian Wu<br>The Second Affiliated Hospital of Zunyi Medical University<br>Zunyi, CHINA
**Keywords:**	Keywords: Anemia; Antioxidant; Composite Dietary Antioxidant Index; cross-sectional study; Dietary.
**Abstract:**	Abstract<br>Background: There is increasing acknowledgment of the potential role that diet rich in antioxidants may play in the prevention of anemia. As a significant indicator of antioxidant-rich diet, the relationship between the composite dietary antioxidant index (CDAI) and anemia has not been extensively studied. Therefore, this study aims to explore the association between CDAI and anemia.<br>Methods: Utilizing datas from the 2003-2018 National Health and Nutrition Examination Survey (NHANES) database. The CDAI was calculated using six dietary antioxidants, based on two 24-hour dietary recall interviews, serving as comprehensive measure of the intake of these antioxidants. Weighted multivariable logistic regression and restricted cubic spline (RCS) analysis was conducted to investigate the association between CDAI and anemia. Furthermore, subgroup analyses were performed to enhance datas reliability.<br>Results: A total of 33914 participants were included in the study, among which 3,416 (10.07%) were diagnosed with anemia. The unadjusted model showed negative association between CDAI and anemia (odds ratio [OR]: 0.94; 95% confidence interval [95%CI]: 0.93 - 0.96; P < 0.001). After adjusting for all covariates , with each increase in CDAI level linked to 3% lower risk of anemia (OR: 0.97; 95%CI: 0.95 - 0.98; P < 0.001). Moreover, when CDAI was categorized into quartiles, the observed trend persisted (P < 0.001). The RCS analysis revealed linear negative relationship between CDAI and anemia (P for nonlinearity = 0.619). Except for sex, smoking, diabetes and hypertension, no statistically significant interactions were found in any subgroup analysis (P < 0.05 for interaction).<br>Conclusion: Our findings suggest that CDAI levels are inversely related to the prevalence of anemia. Consequently, monitoring individuals with low CDAI scores may facilitate the timely identification of anemia and enhance clinical decision-making.
**Order of Authors:**	Qian Wu
	Zhiyu Wang
	Jieyu Xia
	Hui Xu
	Gang Huang
	guangyong Feng
	Xiaoxia Gou
**Response to Reviewers:**	Dear Editors and Reviewers,<br>Thank you for giving us an opportunity to revise our manuscript. On behalf of all the contributing authors, I would like to express our sincere appreciations of your letter and reviewers' constructive comments concerning our article entitled "Association between composite dietary antioxidant indices and anemia: NHANES 2003–2018" (Number: PONE-D-24-28259). Those comments are all valuable and very helpful for revising and

improving our paper. We have studied comments carefully and have made extensive modifications which we hope meet with approval. The editors and reviewers' comments are laid out below in blue font and specific concerns have been numbered. Our responses are provided in normal font, and the specific locations of changes and additions to the manuscript are highlighted in red text. Additionally, we have included grant numbers and funding sources in the funding information section. We have also uploaded the proof of polishing the manuscript. If there are any specific location problems, we will continue to modify.
Thank you and best regards.

Yours sincerely,
Xiaoxia Gou, MD
Department of Head and Neck Oncology
The Second Affiliated Hospital of Zunyi Medical University
Zunyi, Guizhou 563000,China.
E-mail: gouxx2020@163.com

Response to comments from Editors and Reviewers
Editorial comment
1.Comment: Please ensure that your manuscript meets PLOS ONE's style requirements, including those for file naming. The PLOS ONE style templates can be found at
https://journals.plos.org/plosone/s/file?id=wjVg/PLOSOne_formatting_sample_main_body.pdf and
https://journals.plos.org/plosone/s/file?id=ba62/PLOSOne_formatting_sample_title_authors_affiliations.pdf.
1.Response: Thank you for your kind reminder. In response to your suggestions, we revisited the PLOS ONE submission guidelines and implemented several changes, including partial rearrangements of text fonts, figure captions, references, and other elements within the manuscript.

2.Comment: Please ensure that you include a title page within your main document. Wedo appreciate that you have a title page document uploaded as a separate file,however, as per our author guidelines (http://journals.plos.org/plosone/s/submission-guidelines#loc-title-page) we do require this to be part of the manuscript file itself and not uploaded separately. Could you therefore please include the title page into the beginning of your manuscript file itself, listing all authors and affiliations.
2.Response: Thanks for your reminding. We will ensure that title page is included at the beginning of the manuscript.

3.Comment: We note that the grant information you provided in the 'Funding Information' and 'Financial Disclosure' sections do not match. When you resubmit, please ensure that you provide the correct grant numbers for the awards you received for your study in the 'Funding Information' section.
3.Response: We sincerely thank you for your valuable reminding and have added the grant number to the grant information section.
This study was supported by the National Natural Science Foundation of China (82460465); The Natural Science Foundation of Guizhou Province (Qian Ke He Basic Project ZK [2024]346); The Guizhou Anti-Cancer Association Science and Technology Plan Project (Anti-Cancer Association Science and Technology 006[2023]); The Natural Science Foundation of Guizhou Province (Qian Ke He Basic Project ZK [2023]525).

4.Comment: Thank you for stating the following financial disclosure: "1.Guizhou Provincial Department of Science and Technology project 2.Science and Technology Program of Guizhou Anti-Cancer Association" Please state what role the funders took in the study. If the funders had no role, please state: "The funders had no role in study

Powered by Editorial Manager® and ProduXion Manager® from Aries Systems Corporation

design, data collection and analysis, decision to publish, or preparation of the manuscript." If this statement is not correct you must amend it as needed. Please include this amended Role of Funder statement in your cover letter; we will change the online submission form on your behalf.
4.Response: Thank you for your kind reminder. The funder participated in this study, and the specific funding information has been matched and updated in the cover letter. Eg: Xiaoxia Gou: The National Natural Science Foundation of China (82460465); The Natural Science Foundation of Guizhou Province (Qian Ke He Basic Project ZK [2024]346); The Guizhou Anti-Cancer Association Science and Technology Plan Project (Anti-Cancer Association Science and Technology 006[2023]);
Gang Huang: The Natural Science Foundation of Guizhou Province (Qian Ke He Basic Project ZK [2023]525).

5.Comment: Please include a separate caption for each figure in your manuscript.
5.Response: Thanks for your reminding. Each figure in our manuscript is accompanied by distinct figure caption.

6.Comment: Please include your tables as part of your main manuscript and remove the individual files. Please note that supplementary tables (should remain/ be uploaded) as separate "supporting information" files
6.Response: Thank you once again for your kind reminder. We have included the tables as part of the manuscript and ensured that the supplementary tables are uploaded as separate ‘Supporting Information’ files.

Review Comments
Reviewer #1:
1.Comment: This manuscript provides a background on the topic, however, it need a clearer or specific research gab. Additionally, the authors should provide more details on the rationale for selecting the Composite Dietary Antioxidant Index (CDAI). About The CDAI, how to use CDAI, and if you compare it with another method, what kind of comparative advantage that you get.
1.Response: Thank you immensely for your inquiry, your insights are profoundly valuable. Indeed, our introduction section had overlooked critical aspects, particularly a detailed exploration of the CDAI and focus on comparative research. To clearly articulate the research gap and align with your recommendations, we have enriched our introduction section with the following revised content.
1)Based on your recommendations, we have added a section in the Introduction section about specific research gab, with the specifics outlined below:
① (Manuscript, Introduction section, page 3, line 69-72): The prevalence and incidence of anemia have risen significantly, attributed to combination of increased nutrient deficiencies, chronic diseases, inherited hemoglobin disorders, and the use of specific medications [4,5].
Reference:
[4]. Chen S, Xiao J, Cai W, Lu X, Liu C, Dong Y, et al. Association of the systemic immune-inflammation index with anemia: a population-based study. Frontiers in immunology. 2024;15:1391573. http://dx.doi.org/10.3389/fimmu.2024.1391573 PMID: 38799419.
[5].Stevens GA, Paciorek CJ, Flores-Urrutia MC, Borghi E, Namaste S, Wirth JP, et al. National, regional, and global estimates of anaemia by severity in women and children for 2000-19: a pooled analysis of population-representative data. The Lancet Global health. 2022;10(5):e627-e39. http://dx.doi.org/10.1016/s2214-109x(22)00084-5 PMID: 35427520.

② (Manuscript, Introduction section, page 3, line 75-78): Early recognition of anemia presents an opportunity to delay or prevent the onset of the disease and enhance treatment outcomes. Consequently, identifying new indicators closely associated with anemia is of great significance for developing more effective anemia prevention strategies [4].
Reference:
[4]. Chen S, Xiao J, Cai W, Lu X, Liu C, Dong Y, et al. Association of the systemic immune-inflammation index with anemia: a population-based study. Frontiers in

immunology. 2024;15:1391573. http://dx.doi.org/10.3389/fimmu.2024.1391573 PMID: 38799419.

③(Manuscript, Introduction section, page 4, line 91-93): As research enhances our understanding of nutrition and oxidative stress, there is an increasing interest in the role of antioxidant-rich diets in the prevention of anemia.

2) We revised the paper and added necessary descriptions about more details for the CDAI in the introduction section and discussion section. The details are as follows :
① (Manuscript, Introduction section, page 4, line 94-96): The composite dietary antioxidant index (CDAI) was developed by Wright et al as a tool to assess antioxidant intake, serving as a composite score to evaluate dietary antioxidant consumption [16].
Reference:
[16] Wright ME, Mayne ST, Stolzenberg-Solomon RZ, Li Z, Pietinen P, Taylor PR, et al. Development of a comprehensive dietary antioxidant index and application to lung cancer risk in a cohort of male smokers. American journal of epidemiology. 2004;160(1):68-76. http://dx.doi.org/10.1093/aje/kwh173 PMID: 15229119.

② (Manuscript, Introduction section, page 4, line 98-106): Wang et al posited that the CDAI was positively correlated with lower prevalence of chronic kidney disease among American adults [17]. Similarly, Yu et al discovered that elevated CDAI scores were linked to decreased risk of colorectal cancer (CRC) and concluded that food-based antioxidants might contribute to lowering the risk of CRC in the general population [18]. Additionally, another study indicated that higher intake of dietary antioxidants, assessed through the Dietary Antioxidant Quality Score (DAQS) and CDAI, was associated with reduced risk of all-cause and cardiovascular disease mortality in adults with diabetes [19].
Reference:
[17].Wang M, Huang ZH, Zhu YH, He P, Fan QL. Association between the composite dietary antioxidant index and chronic kidney disease: evidence from NHANES 2011-2018. Food & function. 2023;14(20):9279-86. http://dx.doi.org/10.1039/d3fo01157g PMID: 37772927.
[18].Yu YC, Paragomi P, Wang R, Jin A, Schoen RE, Sheng LT, et al. Composite dietary antioxidant index and the risk of colorectal cancer: Findings from the Singapore Chinese Health Study. International journal of cancer. 2022;150(10):1599-608. http://dx.doi.org/10.1002/ijc.33925 PMID: 35001362.
[19].Wang W, Wang X, Cao S, Duan Y, Xu C, Gan D, et al. Dietary Antioxidant Indices in Relation to All-Cause and Cause-Specific Mortality Among Adults With Diabetes: A Prospective Cohort Study. Front Nutr. 2022;9:849727. http://dx.doi.org/10.3389/fnut.2022.849727 PMID: 35600816.

③ (Manuscript, Discussion section on Page 9, line 251-256): In this study, we focus on the CDAI for two primary reasons. Firstly, CDAI serves as a crucial indicator of dietary antioxidant capacity. Unlike single dietary antioxidant indicators, CDAI encompasses key antioxidant nutrients, including vitamin A, vitamin C, vitamin E, zinc, selenium, and carotene, thereby facilitating more comprehensive evaluation of overall diet quality. Secondly, research in related fields has garnered significant attention.

④ (Manuscript, Discussion section on Page 9, line 259-262): Similar, Ma et al in their study on the correlation between CDAI and coronary heart disease, they reported negative correlation, indicating that CDAI is inversely related to the incidence of coronary heart disease (Q4 vs Q1, OR: 0.65, 95%CI: 0.51 - 0.82, P < 0.001) [33].
Reference:
[33].Ma R, Zhou X, Zhang G, Wu H, Lu Y, Liu F, et al. Association between composite dietary antioxidant index and coronary heart disease among US adults: a cross-sectional analysis. BMC public health. 2023;23(1):2426. http://dx.doi.org/10.1186/s12889-023-17373-1 PMID: 38053099.

⑤ (Manuscript, Discussion section, page 10, Line 264-269) : Our study reinforces these findings by showing an inverse relationship between CDAI levels and anemia in American adults (OR: 0.97, 95%CI: 0.96 - 0.98). Specifically, higher CDAI scores were

Powered by Editorial Manager® and ProduXion Manager® from Aries Systems Corporation

linked to lower prevalence of anemia. Our findings align with those of Zhang et al, who reported that among 5,880 participants, higher CDAI was associated with reduced likelihood of renal anemia (adjusted OR: 0.96, 95% CI: 0.94 - 0.98) [34].
Reference:
[34].Zhang L, Yin D, Zhu T, Geng L, Gan L, Ou S, et al. Composite dietary antioxidant index is associated with renal anemia: a cross-sectional study. International urology and nephrology. 2024. http://dx.doi.org/10.1007/s11255-024-04157-8 PMID: 39044023.

⑥ (Manuscript, Discussion section on Page 10, line 279-281): This finding aligns with our results, which show that higher CDAI scores are associated with lower prevalence of anemia, thereby emphasizing the potential therapeutic role of antioxidants.

⑦ (Manuscript, Discussion section on Page 11, line 299-304): However, these studies focused solely on specific antioxidants and did not examine potential synergies among various antioxidant nutrients. Given that our dietary intake typically includes multiple antioxidants, the CDAI seems to exert a comprehensive effect on individuals' pro-antioxidant status and underscores the advantages of thorough assessments of antioxidant exposure [17].
Reference:
[17].Wang M, Huang ZH, Zhu YH, He P, Fan QL. Association between the composite dietary antioxidant index and chronic kidney disease: evidence from NHANES 2011-2018. Food & function. 2023;14(20):9279-86. http://dx.doi.org/10.1039/d3fo01157g PMID: 37772927.

2.Comment: Based on that survey, what kind of food source that lead to increasing level of six nutrients by considering age, sex, race/ethnicity, education level, body mass index (BMI), poverty income ratio (PIR), Please write diet recommendation that is more effective and that is considered as a strategy in anemia prevention (add in your discuss).
2.Response: Based on your recommendations, we have checked the literature carefully and have revised the discussion to include specific dietary recommendations aimed at anemia prevention.
①(Manuscript, Discussion section, page 10, line 273-276): Andrea et al summarize the intricate interactions between antioxidant-rich foods and gut microbiota, inflammation, and obesity, positing that plant-based antioxidant foods, such as vegetables, fruits, and nuts, are essential for health maintenance [36].

② (Manuscript, Discussion section, page 10, line 281-285): Therefore, a balanced diet, such as the Mediterranean diet, along with specific foods like fish, fresh vegetables, and fruits, is recommended for individuals with low CDAI scores [36]. These foods are rich in essential components, including fiber, minerals, vitamins, and antioxidants, which can help prevent the occurrence of anemia.
Reference:
[36].Deledda A, Annunziata G, Tenore GC, Palmas V, Manzin A, Velluzzi F. Diet-Derived Antioxidants and Their Role in Inflammation, Obesity and Gut Microbiota Modulation. Antioxidants (Basel, Switzerland). 2021;10(5). http://dx.doi.org/10.3390/antiox10050708 PMID: 33946864.

③ We have included effective dietary recommendations for the prevention of anemia in the conclusion section. Please refer to the conclusion section, page 12, line 325-331: This study revealed an inverse relationship between CDAI and the prevalence of anemia, with more pronounced effect observed in male nonsmokers and nondiabetic individuals. These findings offer valuable insights for healthcare providers to develop more targeted anemia screening and prevention strategies. Furthermore, Our study suggest the potential role of dietary modifications, particularly for individuals with lower CDAI scores, including the adoption of antioxidant-rich dietary patterns, such as iron supplements and plant-based diets, to help reduce the prevalence of anemia.

Reviewer #2:
1.Comment: How can the 24-hour dietary recall interview method explain the diversity

of daily consumption, how to control consumption on holidays, during parties, when dieting or fasting?

1. Response: Thank you immensely for your inquiry, your insights are profoundly valuable.

1) In line with previous research, participants completed two such interviews. Please refer Manuscript, Method section, page 5, line 137-145: Data on dietary antioxidant intake were obtained from the average of two 24-hour dietary recall interviews conducted as part of the NHANES. The initial dietary recall was performed at mobile examination center (MEC) by trained interviewers who adhered to standardized protocol. During this face-to-face interview, detailed information regarding all food and beverages consumed by participants over the past 24 hours was collected. This was followed by second interview conducted via telephone 3 to 10 days later [23]. Utilizing average dietary intake data from two non-consecutive days is deemed more accurate than relying solely on data from a single day [24].

2) Given the diversity of daily consumption, it may not accurately reflect habitual eating patterns. Acknowledging these limitations, future research could benefit from utilizing multiple food recalls or food frequency questionnaires to obtain a more representative and comprehensive understanding of long-term dietary habits.

Reference:

previous research:

“Ni Y, et al. BMC Public Health. 2024; 24(1):2167. doi: 10.1186/s12889-024-19620-5.”

“Zhu G, et al. Nutrients. 2022; 14(23):5026. doi: 10.3390/nu14235026”

“Wan H, et al. Sci Rep. 2024;14(1):2415. doi: 10.1038/s41598-024-52806-w.”

[23].Pannucci TE, Thompson FE, Bailey RL, Dodd KW, Potischman N, Kirkpatrick SI, et al. Comparing Reported Dietary Supplement Intakes between Two 24-Hour Recall Methods: The Automated Self-Administered 24-Hour Dietary Assessment Tool and the Interview-Administered Automated Multiple Pass Method. Journal of the Academy of Nutrition and Dietetics. 2018;118(6):1080-6.
http://dx.doi.org/10.1016/j.jand.2018.02.013 PMID: 29803270.

[24].Yang T, Yi J, He Y, Zhang J, Li X, Ke S, et al. Associations of Dietary Fats with All-Cause Mortality and Cardiovascular Disease Mortality among Patients with Cardiometabolic Disease. Nutrients. 2022;14(17).
http://dx.doi.org/10.3390/nu14173608 PMID: 36079863.

2.Comment: How to overcome recall bias in this study?

2. Response: Thank you for your professional review. And we clarified that limitation in the discussion section.

We utilized 24-hour dietary recalls to evaluate dietary nutrient intake, relying on self-reported data that may be susceptible to recall bias. We acknowledge this limitation, which is explicitly addressed in our manuscript. (Manuscript, Discussion section, page 11, line 311-313)

3.Comment: How to control that the results of this analysis do not occur by chance, not because of the large number of samples? Are there other dominant factors that can interfere with the relationship between the two, besides the existing variables?

3. Response: Thank you for pointing this out. As you are concerned, several issues need to be addressed, which are replied to in detail as below.

1) This study employed weighted multiple logistic regression analysis to control for confounding variables, thereby enhancing the robustness of our findings.

2) Although this study accounted for numerous clinical variables, potential unmeasured confounders, such as medication use and laboratory indicators (including liver and kidney function, iron levels, vitamins), may still influence CDAI levels and their correlation with outcomes. Consequently, future research should aim to explore more comprehensively the specific associations between these variables. (Manuscript, Discussion section, page 11, line 319-322)

4. Comment: How to improve the results of the relationship between the two, considering that the study design used was cross-sectional?

4. Response: Thanks for your significant reminding. We have included this limitation in the revised manuscript.

The cross-sectional design of this study inherently limits the ability to establish a causal relationship between the variables. Consequently, prospective multicenter studies will

be necessary in the near future to validate our findings. (Manuscript, Discussion section, page 11, line 313-315)

Reviewer #3:
Dear Authors, I appreciate your efforts and hard work. I have a few recommendations.
Comment: Rephrase the opening sentence of the abstract: "Recent studies have revealed that..." This statement requires references. You may report it as there is a documented association between,,,, Add extra details to the method part.
Response: Thank you sincerely for dedicating your time to reviewing our manuscript and for your constructive feedback. We have rephrased the opening sentence of the abstract to state:
(Manuscript, Abstract section, page 2, line 35-36) : There is increasing acknowledgment of the potential role that diet rich in antioxidants may play in the prevention of anemia.

Background
Comment: Please elaborate on the relationship between anemia and antioxidants, and how antioxidants diminish anemia. Protein synthesis? What biological reaction affected by the oxidative stress? What physiological pathway is affect?
Response: We sincerely appreciate the valuable and constructive comments. We have expanded the introduction section to include detailed discussion on the relationship between anemia and antioxidants.
(Manuscript, Introduction section, page 3, line 87-91) Halima et al found that antioxidants exert protective effect against anemia and provide significant alternative benefits for RBC function. This is achieved by preventing lipid peroxidation in RBCs, increasing levels of reduced glutathione (GSH), and reducing RBC permeability [15].
Reference:
[15].Al Balushi H, Hannemann A, Rees D, Brewin J, Gibson JS. The Effect of Antioxidants on the Properties of Red Blood Cells From Patients With Sickle Cell Anemia. Frontiers in physiology. 2019;10:976.
http://dx.doi.org/10.3389/fphys.2019.00976 PMID: 31456691.

Methodology
Comment: i Have concern about the anemia cut score according to smoking status and living in high altitude areas? both affect the Hb cut score? Also you studies only iron deficiency anemia? not all types? clarify and justify why?
Response: Thanks for your significant reminding. We have checked out the relevant information and understand your comments.
1)According to national health industry standards, individuals who smoke or reside at altitudes exceeding 1,000 meters must make adjustments in accordance with the “WS/T 441-2013 Population Anemia Screening Method”. Specifically, for every 1,000 meters of altitude increase in plateau regions, hemoglobin levels should increase by 4%. However, elevation data are not available in the NHANES database. It is important to note that in other comparable NHANES studies, the anemia data utilized were based on laboratory diagnoses, which we recognize as limitation of the current study. We have included this limitation in the revised manuscript. The details are as follows:
(Manuscript, Discussion section, page 11, line 316-318) Thirdly, the anemia data utilized in this study were derived from laboratory diagnoses and may not have accounted for the potential contributions of a history of anemia or biological differences, such as smoking or an increased incidence of thalassemia.
Reference:
“Chen S, et al. Front Immunol. 2024;15:1391573. doi: 10.3389/fimmu.2024.1391573.”
“Wang Y, et al. Ann Hematol. 2023;102(10):2651-2658. doi: 10.1007/s00277-023-05374-4.”
“Drury KE, et al. JAMA Pediatr. 2016;170(1):29-34. doi: 10.1001/jamapediatrics.2015.3065.”
2)This study utilized laboratory-diagnosed anemia data and examined all types of anemia, however, it did not assess laboratory data for iron abnormalities. Consequently, the potential contributions of iron deficiency or other causes of anemia to the incidence reported here remain undetermined, we recognize as limitation of the current study.

Comment: In your exclusion criteria, other than excluding the missing data, do you

	include chronic GI diseases, malabsorption disorders, and pregnancy?<br>Response: Thank you for your professional review. Due to the limited data on chronic gastrointestinal diseases and malabsorption disorders in the NHANES database, as well as references to other anemia-related literature, only pregnant individuals (N = 1,051) were excluded from our study.<br>Please refer to Manuscript, Methods section page 5, line 127-128: After excluding individuals without hemoglobin data (N = 14,320), those lacking CDAI data (N = 4,656), and pregnant participants (N = 1,051).<br>Reference:<br>1.“Csaba P Kovesdy, et al. Clinical Kidney Journal, 2023; 16(2), 303:311. doi: 10.1093/ckj/sfac240.”<br>2.“Chen S, et al. Front Immunol. 2024; 15:1391573. doi: 10.3389/fimmu.2024.1391573”<br><br>The disucsison<br>Comment: It would be beneficial to include earlier research on the relationship between diet consumption and anemia (which are numerous). To support your hypothesis, compare your results with those of these studies.<br>Response: Thank you again for your thorough review and valuable comments. And we have checked the earlier literature carefully and have made the corrections. The details as follows:<br>1)(Manuscript, Discussion section, page 10, line 269-273): In addition, studies focusing on specific anemic populations, such as patients with β- thalassemia, provide further insight into the relationship between anemia severity and antioxidant defenses. Allen et al suggest that the severity of anemia in these patients is linked to depletion of antioxidant defenses, indicating that antioxidant supplementation may be beneficial [35].<br>2)(Manuscript, Discussion section, page 10, line 277-279) : Meanwhile, Jacques et al investigated the impact of various nutrients, including antioxidants like vitamin A and vitamin C, on iron metabolism to mitigate the risk of anemia [37].<br>3)(Manuscript, Discussion section, page 10, line 279-285) : This finding aligns with our results, which show that higher CDAI scores are associated with lower prevalence of anemia, thereby emphasizing the potential therapeutic role of antioxidants. Therefore, a balanced diet, such as the Mediterranean diet, along with specific foods like fish, fresh vegetables, and fruits, is recommended for individuals with low CDAI scores [36]. These foods are rich in essential components, including fiber, minerals, vitamins, and antioxidants, which can help prevent the occurrence of anemia.<br>Reference:<br>[35].Allen A, Perera S, Mettananda S, Rodrigo R, Perera L, Darshana T, et al. Oxidative status in the β-thalassemia syndromes in Sri Lanka; a cross-sectional survey. Free radical biology & medicine. 2021;166:337-47. http://dx.doi.org/10.1016/j.freeradbiomed.2021.02.028 PMID: 33677065.<br>[36].Deledda A, Annunziata G, Tenore GC, Palmas V, Manzin A, Velluzzi F. Diet-Derived Antioxidants and Their Role in Inflammation, Obesity and Gut Microbiota Modulation. Antioxidants (Basel, Switzerland). 2021;10(5). http://dx.doi.org/10.3390/antiox10050708 PMID: 33946864.<br>[37].Berger J, Wieringa FT, Lacroux A, Dijkhuizen MA. Strategies to prevent iron deficiency and improve reproductive health. Nutrition reviews. 2011;69 Suppl 1:S78-86. http://dx.doi.org/10.1111/j.1753-4887.2011.00436.x PMID: 22043887.<br><br>Yours sincerely,<br>Xiaoxia Gou, MD<br>Department of Head and Neck Oncology<br>The Second Affiliated Hospital of Zunyi Medical University<br>Zunyi, Guizhou 563000,China.<br>E-mail: gouxx2020@163.com
**Additional Information:**	
**Question**	**Response**
**Financial Disclosure**	Yes

Powered by Editorial Manager® and ProduXion Manager® from Aries Systems Corporation

Enter a financial disclosure statement that describes the sources of funding for the work included in this submission. Review the submission guidelines for detailed requirements. View published research articles from *PLOS ONE* for specific examples.<br><br>This statement is required for submission and **will appear in the published article** if the submission is accepted. Please make sure it is accurate.<br><br>**Funded studies**<br>Enter a statement with the following details:<br>• Initials of the authors who received each award<br>• Grant numbers awarded to each author<br>• The full name of each funder<br>• URL of each funder website<br>• Did the sponsors or funders play any role in the study design, data collection and analysis, decision to publish, or preparation of the manuscript?<br><br>Did you receive funding for this work?	
Please add funding details.<br>as follow-up to "**Financial Disclosure**<br><br>Enter a financial disclosure statement that describes the sources of funding for the work included in this submission. Review the submission guidelines for detailed requirements. View published research articles from *PLOS ONE* for specific examples.<br><br>This statement is required for submission and **will appear in the published article** if the submission is accepted. Please make sure it is accurate.	This study was supported by the the National Natural Science Foundation of China (82460465); The Natural Science Foundation of Guizhou Province (Qian Ke He Basic Proiect ZK [2024]346); The Guizhou Anti-Cancer Association Science and Technology Plan Project (Anti-Cancer Association Science and Technology 006[2023]).

**Funded studies**<br>Enter a statement with the following details:<br>• Initials of the authors who received each award<br>• Grant numbers awarded to each author<br>• The full name of each funder<br>• URL of each funder website<br>• Did the sponsors or funders play any role in the study design, data collection and analysis, decision to publish, or preparation of the manuscript?<br><br>Did you receive funding for this work?"	
Please select the country of your main research funder (please select carefully as in some cases this is used in fee calculation).<br>as follow-up to "**Financial Disclosure**<br><br>Enter a financial disclosure statement that describes the sources of funding for the work included in this submission. Review the submission guidelines for detailed requirements. View published research articles from *PLOS ONE* for specific examples.<br><br>This statement is required for submission and **will appear in the published article** if the submission is accepted. Please make sure it is accurate.<br><br>**Funded studies**<br>Enter a statement with the following details:<br>• Initials of the authors who received each award<br>• Grant numbers awarded to each author<br>• The full name of each funder<br>• URL of each funder website<br>• Did the sponsors or funders play any role in the study design, data collection and analysis, decision to publish, or preparation of the manuscript?<br><br>Did you receive funding for this work?"	CHINA - CN
**Competing Interests**	The authors have declared that no competing interests exist.

Use the instructions below to enter a competing interest statement for this submission. On behalf of all authors, disclose any competing interests that could be perceived to bias this work—acknowledging all financial support and any other relevant financial or non-financial competing interests.<br><br>This statement is required for submission and **will appear in the published article** if the submission is accepted. Please make sure it is accurate and that any funding sources listed in your Funding Information later in the submission form are also declared in your Financial Disclosure statement.<br><br>View published research articles from *PLOS ONE* for specific examples.<br><br>**NO authors have competing interests**<br><br>Enter: *The authors have declared that no competing interests exist.*<br><br>**Authors with competing interests**<br><br>Enter competing interest details beginning with this statement:<br><br>*I have read the journal's policy and the authors of this manuscript have the following competing interests: [insert competing interests here]*<br><br>* typeset	
**Ethics Statement**<br><br>Enter an ethics statement for this submission. This statement is required if the study involved:<br><br>• Human participants<br>• Human specimens or tissue<br>• Vertebrate animals or cephalopods<br>• Vertebrate embryos or tissues	NA

• Field research<br><br>Write "N/A" if the submission does not require an ethics statement.<br><br>General guidance is provided below. Consult the submission guidelines for detailed instructions. **Make sure that all information entered here is included in the Methods section of the manuscript.**	

Powered by Editorial Manager® and ProduXion Manager® from Aries Systems Corporation

**Format for specific study types**<br><br>**Human Subject Research (involving human participants and/or tissue)**<br>• Give the name of the institutional review board or ethics committee that approved the study<br>• Include the approval number and/or a statement indicating approval of this research<br>• Indicate the form of consent obtained (written/oral) or the reason that consent was not obtained (e.g. the data were analyzed anonymously)<br><br>**Animal Research (involving vertebrate animals, embryos or tissues)**<br>• Provide the name of the Institutional Animal Care and Use Committee (IACUC) or other relevant ethics board that reviewed the study protocol, and indicate whether they approved this research or granted a formal waiver of ethical approval<br>• Include an approval number if one was obtained<br>• If the study involved *non-human primates*, add *additional details* about animal welfare and steps taken to ameliorate suffering<br>• If anesthesia, euthanasia, or any kind of animal sacrifice is part of the study, include briefly which substances and/or methods were applied<br><br>**Field Research**<br><br>Include the following details if this study involves the collection of plant, animal, or other materials from a natural setting:<br>• Field permit number<br>• Name of the institution or relevant body that granted permission	
**Data Availability**<br><br>Authors are required to make all data underlying the findings described fully available, without restriction, and from the time of publication. PLOS allows rare exceptions to address legal and ethical concerns. See the PLOS Data Policy and FAQ for detailed information.	Yes - all data are fully available without restriction

Powered by Editorial Manager® and ProduXion Manager® from Aries Systems Corporation

A Data Availability Statement describing where the data can be found is required at submission. Your answers to this question constitute the Data Availability Statement and **will be published in the article**, if accepted.<br><br>**Important:** Stating 'data available on request from the author' is not sufficient. If your data are only available upon request, select 'No' for the first question and explain your exceptional situation in the text box.<br><br>Do the authors confirm that all data underlying the findings described in their manuscript are fully available without restriction?	
**Describe where the data may be found in full sentences. If you are copying our sample text, replace any instances of XXX with the appropriate details.**<br><br>• If the data are **held or will be held in a public repository**, include URLs, accession numbers or DOIs. If this information will only be available after acceptance, indicate this by ticking the box below. For example: *All XXX files are available from the XXX database (accession number(s) XXX, XXX.*).<br>• If the data are all contained **within the manuscript and/or Supporting Information files**, enter the following: *All relevant data are within the manuscript and its Supporting Information files.*<br>• If neither of these applies but you are able to provide **details of access elsewhere**, with or without limitations, please do so. For example:<br><br>*Data cannot be shared publicly because of [XXX]. Data are available from the XXX Institutional Data Access / Ethics Committee (contact via XXX) for researchers who meet the criteria for access to confidential data.*<br><br>*The data underlying the results presented in the study are available from (include the name of the third party*	Data availability :<br>The National Health and Nutrition Examination Survey (NHANES) database, which is managed by the National Center for Health Statistics (NCHS) of the Centers for Disease Control and Prevention, provided the data used in this study. The authors confirm they did not have any special access privileges that others would not have and others would be able to access these data in the same manner as the authors. The data relevant to this study are publicly available and can be accessed at https://wwwn.cdc.gov/Nchs/Nhanes/2011-2012/CBC_G.htm#LBXHGB. All relevant data from this database have been provided within the paper and its Supporting Information files.

and contact information or URL).<br>• This text is appropriate if the data are owned by a third party and authors do not have permission to share the data.<br><br>* typeset	
Additional data availability information:	

Powered by Editorial Manager® and ProduXion Manager® from Aries Systems Corporation

Manuscript Click here to access/download;Manuscript;Manuscript.docx

Title page

Association between composite dietary antioxidant indices and anemia: NHANES 2003–2018

Qian Wu[1], Zhiyu Wang[2], Jieyu Xia[1], Hui Xu[1], Gang Huang[1], Guangyong Feng[1], Xiaoxia Gou[1*]

1 Department of Head and Neck Oncology, The Second Affiliated Hospital of Zunyi Medical University, Zunyi, Guizhou, China

2 Department of Oncology, The Fifth Affiliated Hospital of Zunyi Medical University, Zhuhai, Guangdong, China

* Email: gouxx2020@163.com

Correspondence Authors:

* Xiaoxia Gou

Department of Head and Neck Oncology, The Second Affiliated Hospital of Zunyi Medical University, Zunyi, Guizhou, China

Email: gouxx2020@163.com

Association between composite dietary antioxidant indices and anemia: NHANES 2003–2018

Abstract

Background: There is increasing acknowledgment of the potential role that diet rich in antioxidants may play in the prevention of anemia. As a significant indicator of antioxidant-rich diet, the relationship between the composite dietary antioxidant index (CDAI) and anemia has not been extensively studied. Therefore, this study aims to explore the association between CDAI and anemia.

Methods: Utilizing datas from the 2003-2018 National Health and Nutrition Examination Survey (NHANES) database. The CDAI was calculated using six dietary antioxidants, based on two 24-hour dietary recall interviews, serving as comprehensive measure of the intake of these antioxidants. Weighted multivariable logistic regression and restricted cubic spline (RCS) analysis was conducted to investigate the association between CDAI and anemia. Furthermore, subgroup analyses were performed to enhance datas reliability.

Results: A total of 33914 participants were included in the study, among which 3,416 (10.07%) were diagnosed with anemia. The unadjusted model showed negative association between CDAI and anemia (odds ratio [OR]: 0.94; 95% confidence interval [95%CI]: 0.93 - 0.96; $P < 0.001$). After adjusting for all covariates , with each increase in CDAI level linked to 3% lower risk of anemia (OR: 0.97; 95%CI: 0.95 - 0.98; $P < 0.001$). Moreover, when CDAI was categorized into quartiles, the observed trend persisted ($P < 0.001$). The RCS analysis revealed linear negative relationship between CDAI and anemia (P for nonlinearity = 0.619). Except for sex, smoking, diabetes and hypertension, no statistically significant interactions were found in any subgroup analysis ($P < 0.05$ for interaction).

Conclusion: Our findings suggest that CDAI levels are inversely related to the prevalence of anemia. Consequently, monitoring individuals with low CDAI scores may facilitate the timely identification of anemia and enhance clinical

decision-making.

Keywords: Anemia; Antioxidant; Composite Dietary Antioxidant Index; Cross-sectional study; Dietary.

Introduction

Anemia is a widespread global health concern that has been consistently linked to various negative outcomes in recent years [1]. It is characterized by an imbalance between the production and destruction of red blood cells (RBCs), resulting in inadequate oxygen delivery to vital tissues like the brain and heart [2]. The diagnosis of anemia typically relies on the hemoglobin level in the blood [3]. The prevalence and incidence of anemia have risen significantly, attributed to combination of increased nutrient deficiencies, chronic diseases, inherited hemoglobin disorders, and the use of specific medications [4, 5]. Anemia can have detrimental effects on cognitive and physical functions [6], leading to decreased economic productivity and higher morbidity and mortality rates [7], presenting significant health challenge in modern society. Early recognition of anemia presents an opportunity to delay or prevent the onset of the disease and enhance treatment outcomes. Consequently, identifying new indicators closely associated with anemia is of great significance for developing more effective anemia prevention strategies [4].

Oxidative stress is defined as an imbalance between the production of reactive oxygen species (ROS) and antioxidant defense mechanisms, and is acknowledged as a major factor in various conditions like inflammation, aging, cancer, and cardiovascular diseases [8]. Numerous studies have emphasized a significant link between oxidative stress and the onset of anemia [9-11]. A modifiable risk factor for reducing oxidative stress is diet [12]. By including dietary antioxidants, individuals can alleviate or prevent oxidative stress-related diseases by neutralizing the harmful effects of ROS [13]. Consistent consumption of antioxidants can reduce oxidative stress levels and enhance the body's ability to withstand it [14]. Halima et al found that antioxidants exert protective effect against anemia and provide significant alternative benefits for RBC function. This is achieved by preventing lipid

peroxidation in RBCs, increasing levels of reduced glutathione (GSH), and reducing RBC permeability [15]. As research enhances our understanding of nutrition and oxidative stress, there is an increasing interest in the role of antioxidant-rich diets in the prevention of anemia.

The composite dietary antioxidant index (CDAI) was developed by Wright et al as a tool to assess antioxidant intake, serving as a composite score to evaluate dietary antioxidant consumption [16]. This index considers key nutrients such as vitamins A, vitamins C, and vitamins E, zinc, selenium, and carotenoids. While previous studies have mainly examined the relationship between CDAI and different diseases. Wang et al posited that the CDAI was positively correlated with lower prevalence of chronic kidney disease among American adults [17]. Similarly, Yu et al discovered that elevated CDAI scores were linked to decreased risk of colorectal cancer (CRC) and concluded that food-based antioxidants might contribute to lowering the risk of CRC in the general population [18]. Additionally, another study indicated that higher intake of dietary antioxidants, assessed through the Dietary Antioxidant Quality Score (DAQS) and CDAI, was associated with reduced risk of all-cause and cardiovascular disease mortality in adults with diabetes [19]. However, the specific connection between CDAI and anemia is yet to be conclusively determined.

A cross-sectional study was conducted using data from the National Health and Nutrition Examination Survey (NHANES) to assess the relationship between CDAI and anemia. The hypothesis posited that higher CDAI scores would correlate with decreased prevalence of anemia.

Materials and methods

NHANES database

The analysis is based on data from the NHANES, a cross-sectional survey conducted by the Centers for Disease Control (CDC) and the National Center for Health Statistics (NCHS). The survey’s objective is to evaluate the health and nutrition status of a representative population, encompassing institutionalized individuals [20]. Since

1999, NHANES has been an ongoing study that releases data every two years [21]. The database is available for free download from the official website. Approval for the study was obtained from the NCHS Research Ethics Review Board, and all participants provided written informed consent, and no external ethic approval was required for this study.

Study population

This study utilized NHANES data spanning from 2003 to 2018, encompassing a total of 80,312 participants. After excluding individuals without hemoglobin data (N = 14,320), those lacking CDAI data (N = 4,656), and pregnant participants (N = 1,051), we further removed cases with missing covariate data. Ultimately, we obtained final sample size of 33914 participants. A flow chart depicting the exclusion criteria is presented in Fig 1.

Exposure and Outcomes

According to World Health Organization (WHO) guidelines, patients with anemia are defined as having hemoglobin (HB) levels below 12 g/dL for women and below 13 g/dL for men [22].

Data on dietary antioxidant intake were obtained from the average of two 24-hour dietary recall interviews conducted as part of the NHANES. The initial dietary recall was performed at mobile examination center (MEC) by trained interviewers who adhered to standardized protocol. During this face-to-face interview, detailed information regarding all food and beverages consumed by participants over the past 24 hours was collected. This was followed by second interview conducted via telephone 3 to 10 days later [23]. Utilizing average dietary intake data from two non-consecutive days is deemed more accurate than relying solely on data from a single day [24]. To evaluate the overall exposure to dietary antioxidants, the intake of six key antioxidants (vitamin A, vitamin C, vitamin E, zinc, selenium, and carotene) was analyzed and quantified using to calculate the CDAI [25]. This involved

standardizing the intake levels of the six antioxidants by subtracting the mean and dividing by the standard deviation, followed by summing up the standardized values:

$$\mathrm{CDAI} = \sum_{i=1}^{n} \left(\frac{x_n - \mu_n}{s_n} \right)$$

Covariates

Potential confounders considered in this study including age, sex, race, education levels, body mass index (BMI), poverty income ratio (PIR), smoking consumption, alcohol consumption, as well as comorbidities like hypertension, diabetes, cancer, and hyperlipidemia. Age was categorized into 40 years, ethnicity into non-Hispanic White, non-Hispanic Black, Mexican American, and others, and education levels into less than high school, high school, and more than high school. Marital status was divided into married/living with partner or widowed/divorced/separated/never married. PIR was categorized as low (□ 2.14) and high (□ 2.14). BMI was categorized as thin/normal (□ 18.5kg/m^2, 18.6 - 24.9kg/m^2), overweight (25.0 - 29.9 kg/m^2), and obese (□ 30 kg/m^2).

Smoking status was categorized into two groups: smokers, defined as those who had smoked at least 100 cigarettes in their lifetime (coded as 'yes'), and non-smokers (coded as 'no') [26]. Alcohol consumption was classified as 'yes' for those who had consumed at least 12 alcoholic drinks in the past year, and 'no' for others [27]. Participants were considered to have a history of diabetes, hypertension, and hyperlipidemia if they reported physicians diagnosis of these conditions. The diagnosis of cancer required meeting two criteria: (1) positive response to 'ever been told they have cancer or a malignancy of any kind' (variable mcq220); (2) providing details for the inquiry 'what kind of cancer?' (variable mcq230A).

Statistical analysis

Participants were categorized into four groups based on quartiles CDAI. Continuous variables were presented as mean ± standard deviation (mean ± SD), while categorical variables were expressed as percentages. The associations between baseline

characteristics and CDAI quartiles were evaluated using chi-square or t tests. Weighted logistic regression model was employed to investigate the relationship between CDAI and anemia, with results reported as adjusted odds ratio (OR) and 95% confidence intervals (CI). Model 1 had no adjustments, while model 2 included adjustments for gender, age, education level, and race. Model 3 further adjusted for PIR, BMI, smoking, drinking, hypertension, diabetes, and hyperlipidemia as covariates in addition to those in Model 2. CDAI was analyzed both as continuous and categorical variable to explore correlations. All statistical analyses were conducted using R 4.3.3 with appropriate weights, and statistical significance was defined as $P < 0.05$.

Ethics statement

The survey procedures are in accordance with the standards outlined in the declaration of Helsinki [28]. All information from the NHANES program is freely available to the public, therefore, the approval of the medical ethics committee board was not required [29].

Results

Baseline characteristics

This study involved 33914 patients who met strict inclusion and exclusion criteria, with 3416 (10.07%) of them diagnosed anemia. The average age of the participants was 47.35 ± 0.22 years. Patients were categorized into 4 groups based on CDAI quartiles, and their baseline characteristics are presented in table 1. Higher GDAI levels were associated with certain characteristics such as younger age, non-Hispanic white married female, non-drinkers, non-smokers, higher BMI, higher PIR, higher education levels, absence of diabetes, cancer, hypertension, and anemia, but presence of hyperlipidemia. Univariable and multivariable logistic analyses showed significant link between CDAI and anemia ($P < 0.001$) (Supplementary Table 1).

The association between CDAI and anemia

Table 2 presents the results of the weighted logistic regression analysis investigating the relationship between anemia and CDAI. The analysis demonstrated robust negative correlation between CDAI and anemia when considering CDAI as continuous variable in model 1 (OR: 0.94; 95%CI: 0.93 - 0.96), model 2 (OR: 0.96; 95%CI: 0.95 - 0.97) and model 3 (OR: 0.97; 95%CI: 0.95 - 0.98). Particularly noteworthy is the significant trend in model 3 (*P* for trend < 0.001) when CDAI was categorized into quartiles, indicating that higher CDAI scores were linked to lower likelihood of anemia. To further confirm this association, restricted cubic spline (RCS) analysis with 3 knots was performed, revealing linear negative correlation between CDAI and anemia (*P* for nonlinearity = 0.619), as depicted in Fig. 2.

The association between antioxidant components and anemia

The relationship between the six antioxidant components of CDAI and anemia was further examined (Table 3), it was observed that vitamin A was linked to anemia in model 1 (OR: 1.00; 95%CI: 1.00 - 1.00, *P* = 0.010). Vitamin C showed an association with anemia in models 1 and 2 (OR: 1.00; 95%CI: 1.00 - 1.00, *P* = 0.030 or *P* = 0.002). After adjusting for all variables, zinc (OR: 0.97; 95% CI: 0.97 - 0.98, *P* < 0.001), vitamin E (OR: 0.98; 95%CI: 0.98 - 0.99, *P* < 0.001), carotene (OR: 1.00; 95% CI:1.00 - 1.00, *P* < 0.001), and selenium (OR: 1.00; 95% CI:1.00 - 1.00, *P* = 0.010) were identified as independent factors associated with anemia.

Subgroup analysis

To further investigate the influence of CDAI on anemia risk within specific subgroups, stratified analysis was performed based on gender, age, race, PIR, BMI, hypertension, diabetes, and hyperlipidemia. Results indicated that except for sex, smoking consumption, and diabetes, there were no significant interactions between CDAI and anemia risk (*P* for interaction > 0.05, Fig. 3). This indicates that the protective effect of CDAI on anemia is particularly pronounced in male non-smokers and non-diabetic

individuals.

Discussion

This study is the first to investigate the relationship between CDAI and the ~~risk~~ prevalence of anemia based on data from NHANES. Upon adjusting for potential confounders, negative association between CDAI and anemia in American adults was observed, with linear trend established through dose-response analysis. Additionally, subgroup analysis indicated the protective effect of CDAI against anemia was particularly significant among male nonsmokers and nondiabetic individuals. Clinically, these results suggest that anemia can be effectively prevented, diagnosed, and treated through appropriate dietary modifications rich in antioxidants.

Diet plays a crucial role in managing the body's oxidative stress levels [30], with dietary antioxidants being essential in lowering the risk of aging, cancer, diabetes, inflammation, liver disease, and cardiovascular disease. The fast-paced lifestyle of modern society leads to an increase in free radicals in the body, which can demage cells, tissues, and organs, ultimately impacting longevity. Antioxidants are key in fighting free radicals, thereby contributing to prevent both short-term and long-term diseases [31]. In this study, we focus on the CDAI for two primary reasons. Firstly, CDAI serves as a crucial indicator of dietary antioxidant capacity. Unlike single dietary antioxidant indicators, CDAI encompasses key antioxidant nutrients, including vitamin A, vitamin C, vitamin E, zinc, selenium, and carotene, thereby facilitating more comprehensive evaluation of overall diet quality. Secondly, research in related fields has garnered significant attention. A study within NHANES demonstrated negative linear correlation between CDAI and hypertension [32]. These results also propose that increasing CDAI levels through a diet rich in antioxidant nutrients could potentially reduce the occurrence of metabolic syndrome [12]. Similar, Ma et al in their study on the correlation between CDAI and coronary heart disease, they reported negative correlation, indicating that CDAI is inversely related to the incidence of coronary heart disease (Q4 vs Q1, OR: 0.65, 95%CI: 0.51 - 0.82, $P < 0.001$) [33]. While research on the relationship between CDAI and anemia is currently limited.

Our study reinforces these findings by showing an inverse relationship between CDAI levels and anemia in American adults (OR: 0.97, 95%CI: 0.96 - 0.98). Specifically, higher CDAI scores were linked to lower prevalence of anemia. Our findings align with those of Zhang et al, who reported that among 5,880 participants, higher CDAI was associated with reduced likelihood of renal anemia (adjusted OR: 0.96, 95% CI: 0.94 - 0.98) [34]. In addition, studies focusing on specific anemic populations, such as patients with β- thalassemia, provide further insight into the relationship between anemia severity and antioxidant defenses. Allen et al suggest that the severity of anemia in these patients is linked to depletion of antioxidant defenses, indicating that antioxidant supplementation may be beneficial [35]. Andrea et al summarize the intricate interactions between antioxidant-rich foods and gut microbiota, inflammation, and obesity, positing that plant-based antioxidant foods, such as vegetables, fruits, and nuts, are essential for health maintenance [36]. Meanwhile, Jacques et al investigated the impact of various nutrients, including antioxidants like vitamin A and vitamin C, on iron metabolism to mitigate the risk of anemia [37]. This finding aligns with our results, which show that higher CDAI scores are associated with lower prevalence of anemia, thereby emphasizing the potential therapeutic role of antioxidants. Therefore, a balanced diet, such as the Mediterranean diet, along with specific foods like fish, fresh vegetables, and fruits, is recommended for individuals with low CDAI scores [36]. These foods are rich in essential components, including fiber, minerals, vitamins, and antioxidants, which can help prevent the occurrence of anemia.

Subsequently, this study explored the correlation between antioxidant components and anemia, discovering that vitamins E, zinc, carotene, and selenium were independently linked to anemia after adjusting for all potential confounders. Vitamin E is recognized as a crucial lipophilic antioxidant in biological membranes, capable of scavenging free radicals and acting as a chain-breaking antioxidant [38]. Severe vitamin E deficiency can lead to impaired immune response and hemolytic anemia caused by free radicals [39]. Sue et al highlighted that the lack of multiple trace element biomarkers (iron, zinc, selenium) was positively associated with anemia

[40]. While Lisa et al showed that low zinc is an independent risk factor for anemia in school-age children and mediates the effect of low selenium on hemoglobin levels [41]. Carotene exert antioxidant effects through direct interactions with free radicals, which occur via electron or hydrogen atom transfer [42]. Additionally, carotene can be converted into vitamin A within the body, promoting iron absorption and metabolism, thereby exerting preventive effect against anemia [43]. However, these studies focused solely on specific antioxidants and did not examine potential synergies among various antioxidant nutrients. Given that our dietary intake typically includes multiple antioxidants, the CDAI seems to exert a comprehensive effect on individuals' pro-antioxidant status and underscores the advantages of thorough assessments of antioxidant exposure [17].

This study has several strengths. Firstly, it is the first cross-sectional survey to explore the relationship between CDAI and anemia, emphasizing the potential impact of dietary antioxidants in reducing anemia prevalence. Secondly, the findings indicate that the consumption of antioxidant- rich foods could potentially decrease the risk of anemia and offer dietary guidance to boost antioxidant intake for individuals with anemia.

However, this study also presents several limitations. Firstly, the dietary data in NHANES is based on self-reporting, which may introduce recall bias, and such errors are unavoidable. Secondly, the cross-sectional design complicates the establishment of causal relationship between CDAI and anemia, indicating that prospective multicenter studies will be necessary in the near future to validate our findings. Thirdly, the anemia data utilized in this study were derived from laboratory diagnoses and may not have accounted for the potential contributions of a history of anemia or biological differences, such as smoking or an increased incidence of thalassemia. Lastly, although this study accounted for numerous clinical variables, potential unmeasured confounders, such as medication use and laboratory indicators, including liver and kidney function, iron levels, and vitamins, may still influence CDAI levels and their correlation with outcomes. Consequently, future research should aim to explore the specific associations between these variables more comprehensively.

Conclusion

This study revealed an inverse relationship between CDAI and the prevalence of anemia, with more pronounced effect observed in male nonsmokers and nondiabetic individuals. These findings offer valuable insights for healthcare providers to develop more targeted anemia screening and prevention strategies. Furthermore, Our study suggest the potential role of dietary modifications, particularly for individuals with lower CDAI scores, including the adoption of antioxidant-rich dietary patterns, such as iron supplements and plant-based diets, to help reduce the prevalence of anemia. Additionally, further large-scale prospective studies are necessary to confirm these findings.

Reference

[1]. Lucca U, Tettamanti M, Tiraboschi P, Logroscino G, Landi C, Sacco L, et al. Incidence of dementia in the oldest-old and its relationship with age: The Monzino 80-plus population-based study. Alzheimer's & dementia : the journal of the Alzheimer's Association. 2020;16(3):472-81. http://dx.doi.org/10.1016/j.jalz.2019.09.083 PMID: 31786127.

[2]. Kassebaum NJ. The Global Burden of Anemia. Hematology/oncology clinics of North America. 2016;30(2):247-308. http://dx.doi.org/10.1016/j.hoc.2015.11.002 PMID: 27040955.

[3]. Bansal PG, Toteja GS, Bhatia N, Gupta S, Kaur M, Adhikari T, et al. Comparison of haemoglobin estimates using direct & indirect cyanmethaemoglobin methods. The Indian journal of medical research. 2016;144(4):566-71. http://dx.doi.org/10.4103/0971-5916.200882 PMID: 28256465.

[4]. Chen S, Xiao J, Cai W, Lu X, Liu C, Dong Y, et al. Association of the systemic immune-inflammation index with anemia: a population-based study. Frontiers in immunology. 2024;15:1391573. http://dx.doi.org/10.3389/fimmu.2024.1391573 PMID: 38799419.

[5]. Stevens GA, Paciorek CJ, Flores-Urrutia MC, Borghi E, Namaste S, Wirth JP, et al. National, regional, and global estimates of anaemia by severity in women and children for 2000-19: a pooled analysis of population-representative data. The Lancet Global health. 2022;10(5):e627-e39. http://dx.doi.org/10.1016/s2214-109x(22)00084-5 PMID: 35427520.

[6]. Kinyoki D, Osgood-Zimmerman AE, Bhattacharjee NV, Kassebaum NJ, Hay SI. Anemia prevalence in women of reproductive age in low- and middle-income countries between 2000 and 2018. Nature medicine. 2021;27(10):1761-82. http://dx.doi.org/10.1038/s41591-021-01498-0 PMID: 34642490.

[7]. Martinsson A, Andersson C, Andell P, Koul S, Engström G, Smith JG. Anemia in the general population: prevalence, clinical correlates and prognostic impact. European journal of epidemiology. 2014;29(7):489-98. http://dx.doi.org/10.1007/s10654-014-9929-9 PMID: 24952166.

[8]. Du W, Adam Z, Rani R, Zhang X, Pang Q. Oxidative stress in Fanconi anemia hematopoiesis and disease progression. Antioxidants & redox signaling. 2008;10(11):1909-21. http://dx.doi.org/10.1089/ars.2008.2129 PMID: 18627348.

[9]. Meroño T, Dauteuille C, Tetzlaff W, Martín M, Botta E, Lhomme M, et al. Oxidative stress, HDL functionality and effects of intravenous iron administration in women with iron deficiency anemia. Clinical nutrition (Edinburgh, Scotland). 2017;36(2):552-8. http://dx.doi.org/10.1016/j.clnu.2016.02.003 PMID: 26926576.

[10]. Castilhos LG, de Oliveira JS, Adefegha SA, Magni LP, Doleski PH, Abdalla FH, et al. Increased oxidative stress alters nucleosides metabolite levels in sickle cell anemia. Redox report : communications in free radical research. 2017;22(6):451-9. http://dx.doi.org/10.1080/13510002.2017.1288973 PMID: 28209096.

[11]. Podkowińska A, Formanowicz D. Chronic Kidney Disease as Oxidative Stress- and Inflammatory-Mediated Cardiovascular Disease. Antioxidants (Basel, Switzerland). 2020;9(8). http://dx.doi.org/10.3390/antiox9080752 PMID: 32823917.

[12]. Zhu Y, Bing J, Zheng L, Hao P, Teng X, Wan L. The association between composite dietary antioxidant index and the metabolic syndrome: NHANES 2007–2018. Journal of Functional Foods. 2024;116:106200. http://dx.doi.org/https://doi.org/10.1016/j.jff.2024.106200 PMID:

[13]. Corrochano AR, Buckin V, Kelly PM, Giblin L. Invited review: Whey proteins as antioxidants and promoters of cellular antioxidant pathways. Journal of dairy science. 2018;101(6):4747-61. http://dx.doi.org/10.3168/jds.2017-13618 PMID: 29605324.

[14]. Ogura Y, Kitada M, Koya D. Sirtuins and Renal Oxidative Stress. Antioxidants (Basel, Switzerland). 2021;10(8). http://dx.doi.org/10.3390/antiox10081198 PMID: 34439446.

[15]. Al Balushi H, Hannemann A, Rees D, Brewin J, Gibson JS. The Effect of Antioxidants on the Properties of Red Blood Cells From Patients With Sickle Cell Anemia. Frontiers in physiology. 2019;10:976. http://dx.doi.org/10.3389/fphys.2019.00976 PMID: 31456691.

[16]. Wright ME, Mayne ST, Stolzenberg-Solomon RZ, Li Z, Pietinen P, Taylor PR, et al. Development of a comprehensive dietary antioxidant index and application to lung cancer risk in a cohort of male smokers. American journal of epidemiology. 2004;160(1):68-76. http://dx.doi.org/10.1093/aje/kwh173 PMID: 15229119.

[17]. Wang M, Huang ZH, Zhu YH, He P, Fan QL. Association between the composite dietary antioxidant index and chronic kidney disease: evidence from NHANES 2011-2018. Food & function. 2023;14(20):9279-86. http://dx.doi.org/10.1039/d3fo01157g PMID: 37772927.

[18]. Yu YC, Paragomi P, Wang R, Jin A, Schoen RE, Sheng LT, et al. Composite dietary antioxidant index and the risk of colorectal cancer: Findings from the Singapore Chinese Health Study. International journal of cancer. 2022;150(10):1599-608. http://dx.doi.org/10.1002/ijc.33925 PMID: 35001362.

[19]. Wang W, Wang X, Cao S, Duan Y, Xu C, Gan D, et al. Dietary Antioxidant Indices in Relation to All-Cause and Cause-Specific Mortality Among Adults With Diabetes: A Prospective Cohort Study. Front Nutr. 2022;9:849727. http://dx.doi.org/10.3389/fnut.2022.849727 PMID: 35600816.

[20]. Qing L, Zhu Y, Yu C, Zhang Y, Ni J. Exploring the association between dietary Inflammatory Index and chronic pain in US adults using NHANES 1999-2004. Scientific reports. 2024;14(1):8726. http://dx.doi.org/10.1038/s41598-024-58030-w PMID: 38622145.

[21]. Scinicariello F, Buser MC, Feroe AG, Attanasio R. Antimony and sleep-related disorders: NHANES 2005-2008. Environmental research. 2017;156:247-52. http://dx.doi.org/10.1016/j.envres.2017.03.036 PMID: 28363141.

[22]. Safiri S, Kolahi AA, Noori M, Nejadghaderi SA, Karamzad N, Bragazzi NL, et al. Burden of anemia and its underlying causes in 204 countries and territories, 1990-2019: results from the Global Burden of Disease Study 2019. Journal of hematology & oncology. 2021;14(1):185. http://dx.doi.org/10.1186/s13045-021-01202-2 PMID: 34736513.

[23]. Pannucci TE, Thompson FE, Bailey RL, Dodd KW, Potischman N, Kirkpatrick SI, et al. Comparing Reported Dietary Supplement Intakes between Two 24-Hour Recall Methods: The Automated Self-Administered 24-Hour Dietary Assessment Tool and the Interview-Administered Automated Multiple Pass Method. Journal of the Academy of Nutrition and Dietetics. 2018;118(6):1080-6. http://dx.doi.org/10.1016/j.jand.2018.02.013 PMID: 29803270.

[24]. Yang T, Yi J, He Y, Zhang J, Li X, Ke S, et al. Associations of Dietary Fats with All-Cause Mortality and Cardiovascular Disease Mortality among Patients with Cardiometabolic Disease. Nutrients. 2022;14(17). http://dx.doi.org/10.3390/nu14173608 PMID: 36079863.

[25]. Xu Q, Qian X, Sun F, Liu H, Dou Z, Zhang J. Independent and joint associations of dietary antioxidant intake with risk of post-stroke depression and all-cause mortality. Journal of affective disorders. 2023;322:84-90. http://dx.doi.org/10.1016/j.jad.2022.11.013 PMID: 36372128.

[26]. Wang MY, Li J, Peng HY, Liu J, Huang KL, Li L, et al. Patients with different types of arthritis may be at risk for major depression: results from the National Health and Nutrition Examination Survey 2007-2018. Annals of palliative medicine. 2021;10(5):5280-8. http://dx.doi.org/10.21037/apm-21-279 PMID: 33977741.

[27]. King DE, Xiang J. The Dietary Inflammatory Index Is Associated With Diabetes Severity. Journal of the American Board of Family Medicine : JABFM. 2019;32(6):801-6. http://dx.doi.org/10.3122/jabfm.2019.06.190092 PMID: 31704748.

[28]. Xu H, Wen Q, Xing X, Chen Y, Zhu Q, Tan M, et al. High Dietary Inflammatory Index increases the risk of female infertility: An analysis of NHANES 2013-2018. Nutrition research (New York, NY). 2024;125:50-60. http://dx.doi.org/10.1016/j.nutres.2024.02.006 PMID: 38503022.

[29]. Luo J, Chen T, Chen Y, Huang ZM, Li XJ, Chen HK, et al. The association between homocysteine and bacterial vaginosis: results from NHANES 2001-2004. Scientific reports. 2023;13(1):21388. http://dx.doi.org/10.1038/s41598-023-45494-5 PMID: 38049434.

[30]. Teng TQ, Liu J, Hu FF, Li QQ, Hu ZZ, Shi Y. Association of composite dietary antioxidant index with prevalence of stroke: insights from NHANES 1999-2018. Frontiers in immunology. 2024;15:1306059. http://dx.doi.org/10.3389/fimmu.2024.1306059 PMID: 38524123.

[31]. Neha K, Haider MR, Pathak A, Yar MS. Medicinal prospects of antioxidants: A review. European journal of medicinal chemistry. 2019;178:687-704. http://dx.doi.org/10.1016/j.ejmech.2019.06.010 PMID: 31228811.

[32]. Wu M, Si J, Liu Y, Kang L, Xu B. Association between composite dietary antioxidant index and hypertension: insights from NHANES. Clinical and experimental hypertension (New

York, NY : 1993). 2023;45(1):2233712. http://dx.doi.org/10.1080/10641963.2023.2233712 PMID: 37439538.

[33]. Ma R, Zhou X, Zhang G, Wu H, Lu Y, Liu F, et al. Association between composite dietary antioxidant index and coronary heart disease among US adults: a cross-sectional analysis. BMC public health. 2023;23(1):2426. http://dx.doi.org/10.1186/s12889-023-17373-1 PMID: 38053099.

[34]. Zhang L, Yin D, Zhu T, Geng L, Gan L, Ou S, et al. Composite dietary antioxidant index is associated with renal anemia: a cross-sectional study. International urology and nephrology. 2024. http://dx.doi.org/10.1007/s11255-024-04157-8 PMID: 39044023.

[35]. Allen A, Perera S, Mettananda S, Rodrigo R, Perera L, Darshana T, et al. Oxidative status in the β-thalassemia syndromes in Sri Lanka; a cross-sectional survey. Free radical biology & medicine. 2021;166:337-47. http://dx.doi.org/10.1016/j.freeradbiomed.2021.02.028 PMID: 33677065.

[36]. Deledda A, Annunziata G, Tenore GC, Palmas V, Manzin A, Velluzzi F. Diet-Derived Antioxidants and Their Role in Inflammation, Obesity and Gut Microbiota Modulation. Antioxidants (Basel, Switzerland). 2021;10(5). http://dx.doi.org/10.3390/antiox10050708 PMID: 33946864.

[37]. Berger J, Wieringa FT, Lacroux A, Dijkhuizen MA. Strategies to prevent iron deficiency and improve reproductive health. Nutrition reviews. 2011;69 Suppl 1:S78-86. http://dx.doi.org/10.1111/j.1753-4887.2011.00436.x PMID: 22043887.

[38]. Takahashi M, Tsuchiya J, Niki EJAotNYAoS. Mode of Action of Vitamin E as Antioxidant in the Membranes as Studied by Spin Labeling. 1989;570. PMID:

[39]. Jilani T, Iqbal MP. Vitamin E deficiency in South Asian population and the therapeutic use of alpha-tocopherol (Vitamin E) for correction of anemia. Pakistan journal of medical sciences. 2018;34(6):1571-5. http://dx.doi.org/10.12669/pjms.346.15880 PMID: 30559825.

[40]. MacDonell SO, Miller JC, Harper MJ, Reid MR, Haszard JJ, Gibson RS, et al. Multiple Micronutrients, Including Zinc, Selenium and Iron, Are Positively Associated with Anemia in New Zealand Aged Care Residents. Nutrients. 2021;13(4). http://dx.doi.org/10.3390/nu13041072 PMID: 33806205.

[41]. Houghton LA, Parnell WR, Thomson CD, Green TJ, Gibson RS. Serum Zinc Is a Major Predictor of Anemia and Mediates the Effect of Selenium on Hemoglobin in School-Aged Children in a Nationally Representative Survey in New Zealand. The Journal of nutrition. 2016;146(9):1670-6. http://dx.doi.org/10.3945/jn.116.235127 PMID: 27466609.

[42]. Zhao M, Zhang D, Zhang Q, Lin Y, Cao H. Association between composite dietary antioxidant index and hyperlipidemia: a cross-sectional study from NHANES (2005-2020). Scientific reports. 2024;14(1):15935. http://dx.doi.org/10.1038/s41598-024-66922-0 PMID: 38987566.

[43]. Cañete A, Cano E, Muñoz-Chápuli R, Carmona R. Role of Vitamin A/Retinoic Acid in Regulation of Embryonic and Adult Hematopoiesis. Nutrients. 2017;9(2). http://dx.doi.org/10.3390/nu9020159 PMID: 28230720.

Table 1. The weighted baseline characteristics by CDAI quartiles.

Characteristics	Total (N = 33914)	Quartiles of CDAI				*P* value
		Q1 (N = 8482)	Q2 (N = 8477)	Q3 (N = 8477)	Q4 (N = 8478)	
**Age (years)**	47.35 ± 0.22	47.35 ± 0.22	47.84 ± 0.30	47.99 ± 0.26	47.66 ± 0.32	**< 0.001**
**Sex**						**< 0.001**
Female	17110 (50.45)	4622 (54.49)	4182 (49.33)	4175 (49.25)	4131 (48.73)	
Male	16804 (49.55)	3860 (45.51)	4295 (50.67)	4302 (50.75)	4347 (51.27)	
**Race**						**< 0.001**
Non-Hispanic black	6977 (20.57)	2124 (25.04)	1712 (20.20)	1546 (18.24)	1595 (18.81)	
Non-Hispanic white	15510 (45.73)	3555 (41.91)	3921 (46.25)	4070 (48.01)	3964 (46.76)	
Mexican American	5323 (15.70)	1287 (15.17)	1368 (16.14)	1352 (15.95)	1316 (15.52)	
Other Hispanic	6104 (18.00)	2124 (25.04)	1476 (17.41)	1509 (17.80)	1603 (18.91)	
**Education levels**						**< 0.001**
High school	4704 (13.87)	1471 (17.34)	1207 (14.24)	1067 (12.59)	959 (11.31)	
Less than high school	3467 (10.22)	1221 (14.40)	909 (10.72)	748 (8.82)	589 (6.95)	
More than high school	25743 (75.91)	5790 (68.26)	6361 (75.04)	6662 (78.59)	6930 (81.74)	
**Marital status**						**< 0.001**
Divorced	3763 (11.10)	1098 (12.95)	899 (10.61)	889 (10.61)	877 (10.34)	
Living with partner	2660 (7.84)	677 (7.98)	611 (7.21)	642 (7.57)	730 (8.61)	
Married	17708 (52.21)	3971 (46.82)	4541 (53.57)	4653 (54.89)	4543 (53.59)	
Never married	5905 (17.42)	1549 (18.26)	1416 (16.70)	1378 (16.26)	1562 (18.42)	
Separated/Windowed	3878 (11.43)	1187 (13.99)	1010 (11.91)	915 (10.79)	766 (9.04)	
**Drinking consumption**						**< 0.001**
No	24345 (71.78)	6494 (72.88)	6184 (72.95)	5874 (69.29)	5793 (68.37)	
Yes	9569 (28.22)	1988 (27.12)	2293 (27.05)	2603 (30.71)	2685 (33.83)	
**Smoking consumption**						**< 0.001**
No	18318 (54.01)	4204 (49.56)	4537 (53.52)	4716 (55.63)	4861 (57.34)	
Yes	15596 (45.99)	4278 (50.44)	3940 (46.48)	3761 (44.37)	3617 (42.66)	
**BMI**						**< 0.001**
Normal	9255 (27.29)	2249 (26.51)	2218 (26.17)	2263 (26.70)	2525 (29.78)	
Thin	518 (1.53)	156 (1.84)	110 (1.30)	113 (1.33)	139 (1.64)	
Obese	12865 (37.93)	3312 (39.05)	3247 (38.30)	3229 (38.09)	3077 (36.30)	
Overweight	11276 (33.25)	2765 (32.60)	2902 (34.23)	2872 (33.88)	2737 (32.28)	

**PIR**						**< 0.001**
≥2.14	16988 (50.09)	3426 (40.39)	4192 (49.45)	4643 (54.77)	4727 (55.76)	
<2.14	16926 (49.91)	5056 (59.61)	4285 (50.55)	3834 (45.23)	3751 (44.24)	
**Hypertension**						**< 0.001**
No	19345 (57.04)	4463 (52.62)	4711 (55.57)	4972 (58.65)	5199 (61.32)	
Yes	14569 (42.96)	4019 (47.38)	3766 (44.43)	3505 (41.35)	3279 (38.68)	
**Hyperlipidemia**						**< 0.001**
No	9725 (28.68)	2237 (26.37)	2331 (27.50)	2443 (28.82)	2714 (32.01)	
Yes	24189 (71.32)	6245 (73.62)	6146 (72.50)	6034 (71.18)	5764 (67.99)	
**Diabetes**						**< 0.001**
Borderline	746 (2.20)	181 (2.13)	192 (2.26)	179 (2.11)	194 (2.29)	
No	28883 (85.17)	7051 (83.13)	7112 (83.90)	7306 (86.19)	7414 (87.45)	
Yes	4285 (12.63)	1250 (14.74)	1173 (13.84)	992 (11.70)	870 (10.26)	
**Anemia**						**< 0.001**
No	30498 (89.93)	7440 (87.72)	7589 (89.52)	7655 (90.30)	7814 (92.17)	
Yes	3416 (10.07)	1042 (12.28)	888 (10.48)	822 (9.70)	664 (7.83)	
**Cancer**						**0.030**
No	30678 (90.46)	7681 (90.56)	7590 (89.54)	7662 (90.39)	7745 (91.35)	
Yes	3236 (9.54)	801 (9.44)	887 (10.46)	815 (9.61)	733 (8.65)	

The first quartile of CDAI is defined as Q1, with the subsequent quartiles being defined as Q2, Q3, and Q4. Note: Data are presented as mean (SD) or n (%). BMI, body mass index; HB, hemoglobin; PIR, poverty income ratio; CDAI, composite dietary antioxidant indexes. *P* values < 0.05 are in bold.

Table 2. The association between CDAI and anemia.

Exposure	Model 1		Model 2		Model 3	
	OR [95%CI]	*P*	OR [95%CI]	*P*	OR [95%CI]	*P*
CDAI	0.94 (0.93,0.96)	**<0.001**	0.96 (0.95,0.97)	**<0.001**	0.97 (0.95,0.98)	**<0.001**
CDAIQ						
Q1	ref		ref		ref	
Q2	0.85 (0.75,0.97)	**0.010**	0.98 (0.86,1.11)	0.690	0.99 (0.86,1.13)	0.870
Q3	0.74 (0.65,0.84)	**<0.001**	0.89 (0.78,1.02)	0.140	0.93 (0.81,1.07)	0.490
Q4	0.56 (0.49,0.64)	**<0.001**	0.68 (0.59,0.77)	**<0.001**	0.71 (0.62,0.81)	**<0.001**
P for trend		**<0.001**		**<0.001**		**<0.001**

Model 1: no covariates were adjusted.

Model 2: adjusted for gender, age, education level and race.
Model 3: adjusted for gender, age, race, education level, PIR, BMI, smoke, drink, hypertension, diabetes and hyperlipidemia. *P* values < 0.05 are in bold.

Table 3. The weighted logistic regression analysis of the association between antioxidant components and anemia.

antioxidant components	Model 1		Model 2		Model 3	
	OR [95%CI]	*P*	OR [95%CI]	*P*	OR [95%CI]	*P*
Vitamins A (mg)	1.00 (1.00,1.00)	**0.010**	1.00 (1.00,1.00)	0.890	1.00 (1.00,1.00)	0.910
Vitamins C (mg)	1.00 (1.00,1.00)	**0.030**	1.00 (1.00,1.00)	**0.020**	1.00 (1.00,1.00)	0.050
Vitamins E (mg)	0.96 (0.95,0.97)	**<0.001**	0.98 (0.97,0.99)	**< 0.001**	0.98 (0.98,0.99)	**<0.001**
Zinc (mg)	0.94 (0.94,0.95)	**<0.001**	0.97 (0.96,0.98)	**<0.001**	0.97 (0.97,0.98)	**<0.001**
Selenium (mg)	0.99 (0.99,1.00)	**<0.001**	1.00 (1.00,1.00)	**<0.001**	1.00 (1.00,1.00)	**<0.001**
Carotene (mg)	1.00 (1.00,1.00)	**<0.001**	1.00 (1.00,1.00)	**0.001**	1.00 (1.00,1.00)	**0.010**

Model 1: no covariates were adjusted.
Model 2: adjusted for gender, age, education level and race.
Model 3: adjusted for gender, age, race, education level, PIR, BMI, smoke, drink, hypertension, diabetes and hyperlipidemia. *P* values < 0.05 are in bold.

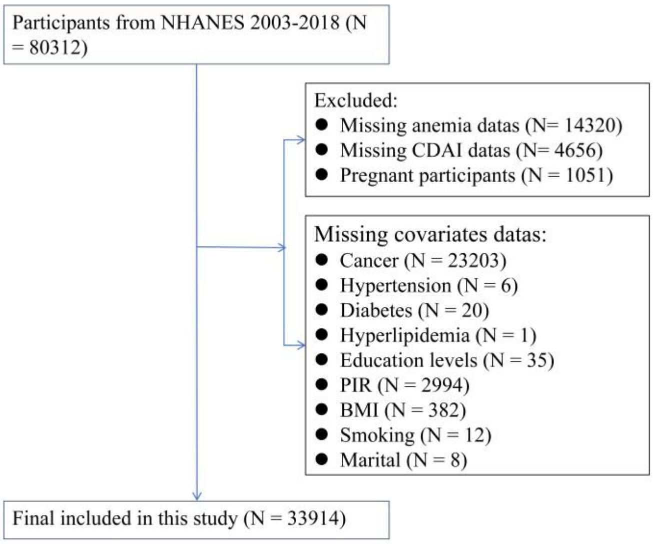

Fig 1. **Flow chart of participant selection in NHANES.** Abbreviations: NHANES, National Health

and Nutrition Examination Survey; CDAI, Composite Dietary Antioxidant Index

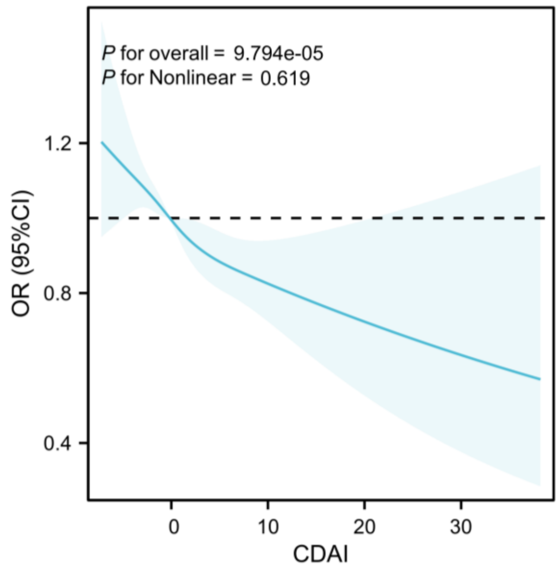

Fig 2. The RCS curve of the association between CDAI and anemia. RCS regression was adjusted by model 3. RCS, restricted cubic spline; CDAI, composite dietary antioxidant index; OR, odds ratio.

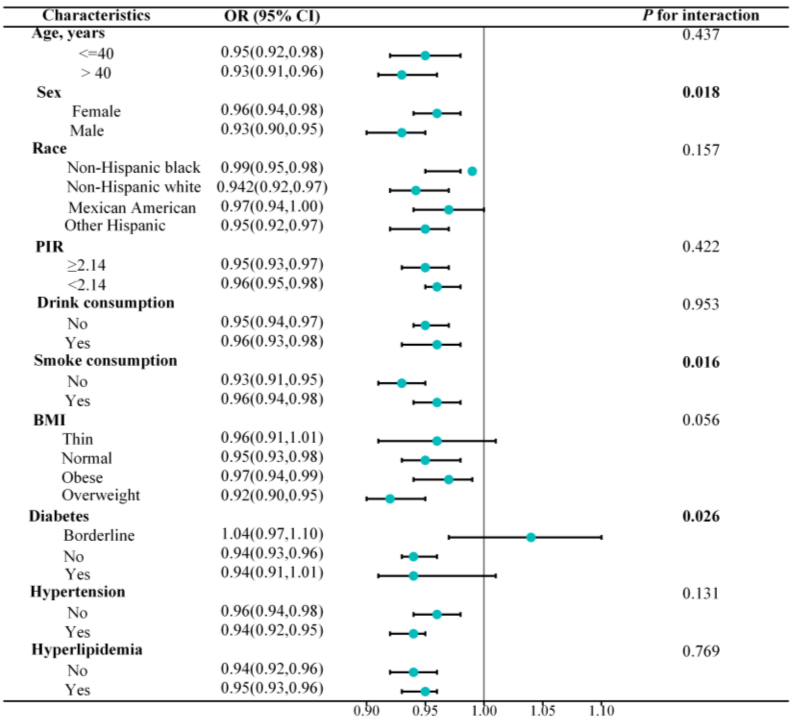

Fig 3. **Subgroups analyses for the association between CDAI and anemia.** BMI, body mass index; PIR, poverty income ratio.

 Click here to access/download;Figure;Figure 1.tif

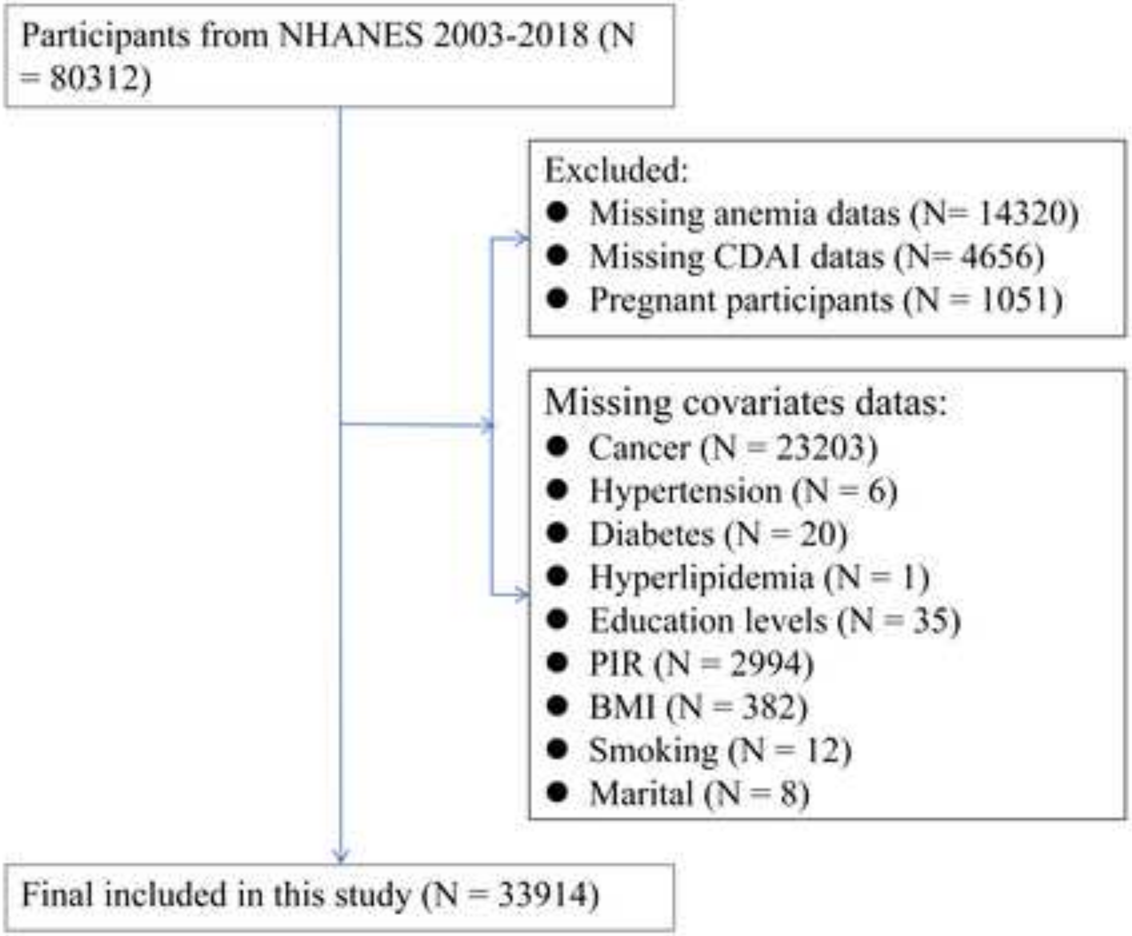

Click here to access/download;Figure;Figure 2.tif

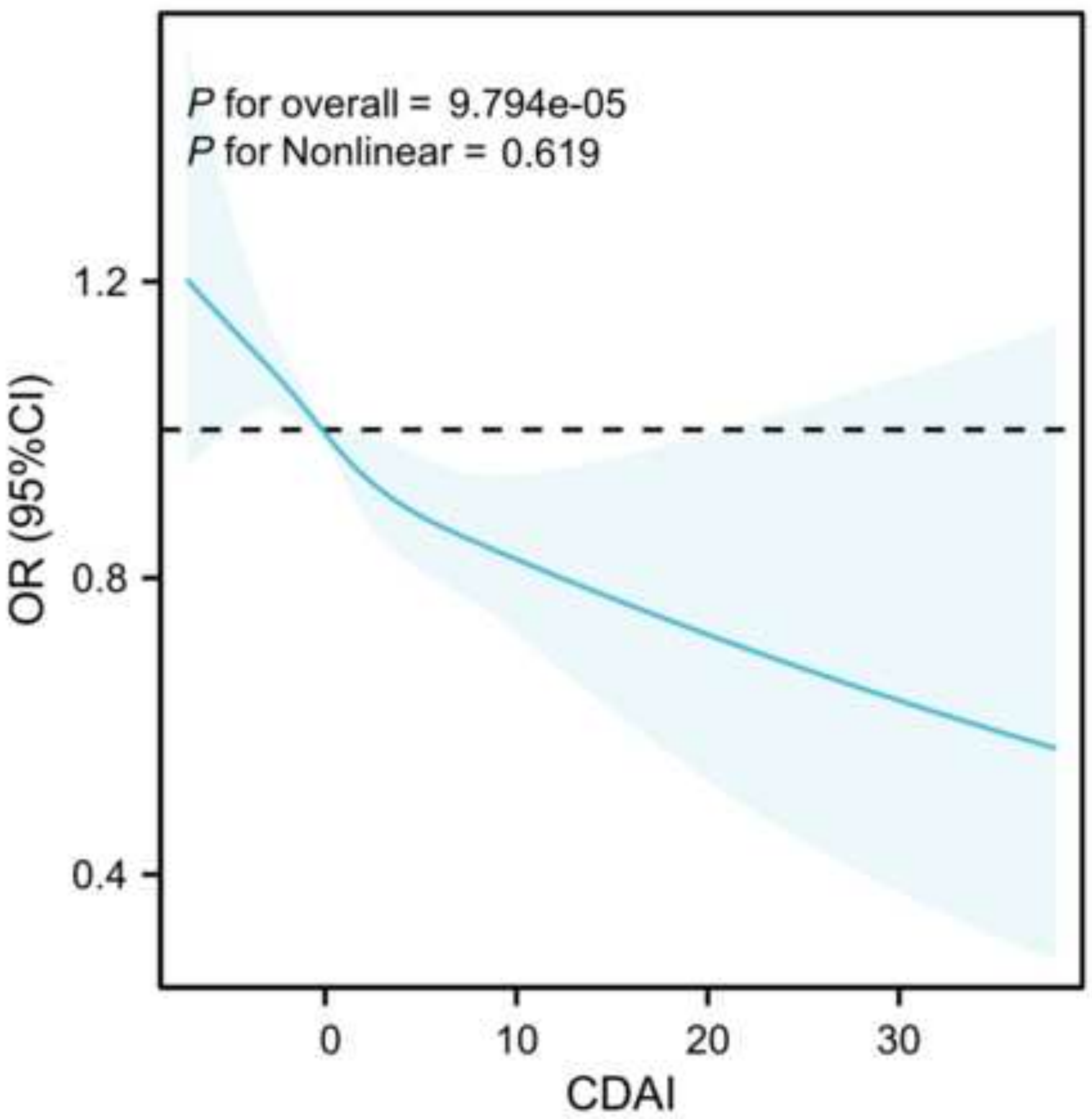

rev. Figure 3

Click here to access/download;Figure;Figure 3.tif

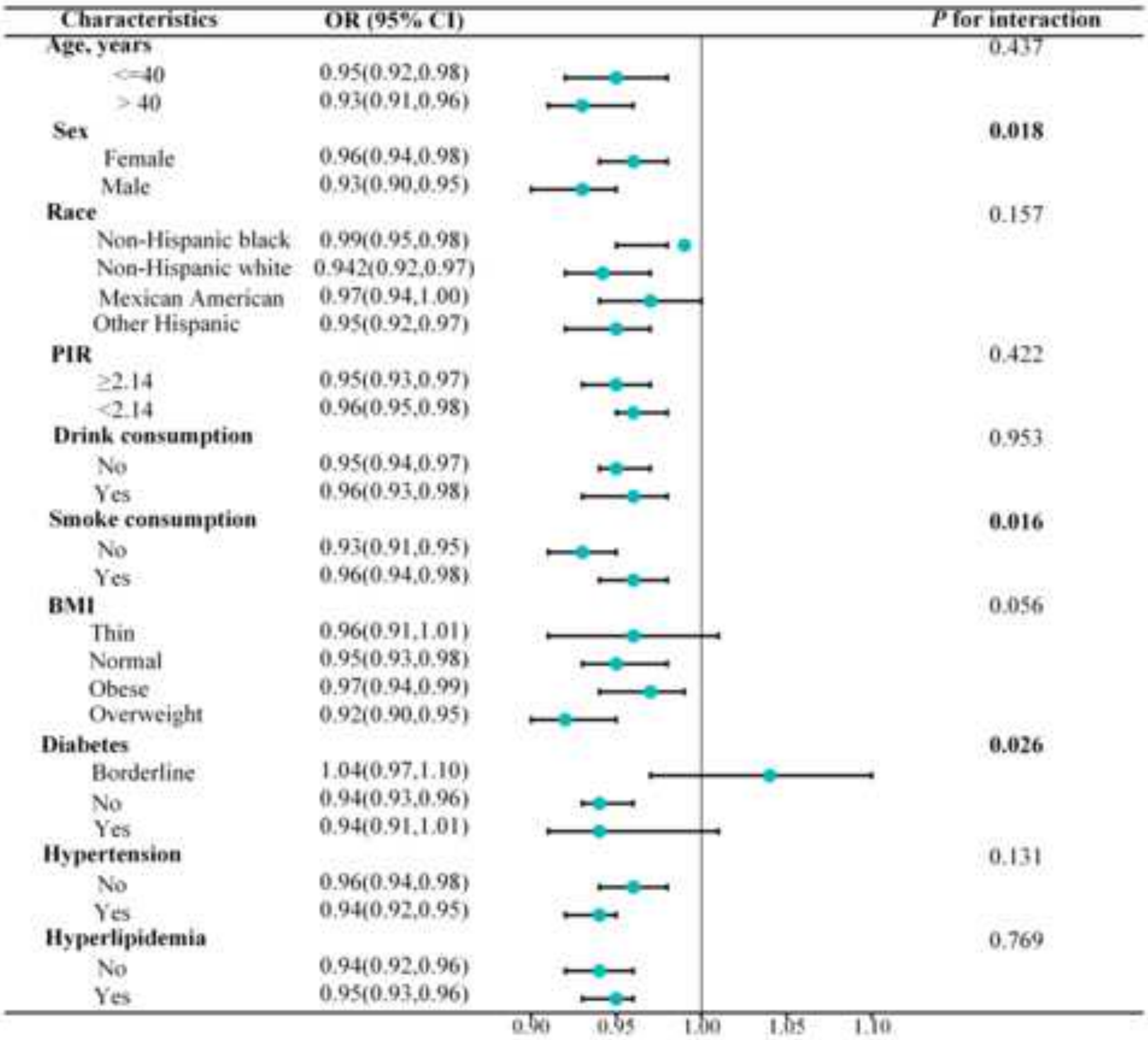

code

Click here to access/download
Supporting Information
code.txt

Raw datas

Click here to access/download
Supporting Information
Raw datas.xlsx

Title page

Association between composite dietary antioxidant indices and anemia: NHANES 2003–2018

Qian Wu[1], Zhiyu Wang[2], Jieyu Xia[1], Hui Xu[1], Gang Huang[1], Guangyong Feng[1], Xiaoxia Gou[1*]

1 Department of Head and Neck Oncology, The Second Affiliated Hospital of Zunyi Medical University, Zunyi, Guizhou, China

2 Department of Oncology, The Fifth Affiliated Hospital of Zunyi Medical University, Zhuhai, Guangdong, China

***** Email: gouxx2020@163.com

Correspondence Authors:

***** Xiaoxia Gou

Department of Head and Neck Oncology, The Second Affiliated Hospital of Zunyi Medical University, Zunyi, Guizhou, China

Email: gouxx2020@163.com

Association between composite dietary antioxidant indices and anemia: NHANES 2003–2018

Abstract

Background: ~~Recent studies have revealed that a diet high in antioxidants could play a role in preventing anemia.~~ ~~The composite dietary antioxidant index (CDAI) is used to measure antioxidant intake. Nevertheless, the exact link between CDAI and anemia remains uncertain~~. There is increasing acknowledgment of the potential role that diet rich in antioxidants may play in the prevention of anemia. As a significant indicator of antioxidant-rich diet, the relationship between the composite dietary antioxidant index (CDAI) and anemia has not been extensively studied. Therefore, this study aims to explore the association between CDAI and anemia.

Methods: Utilizing datas from the 2003-2018 National Health and Nutrition Examination Survey (NHANES) database. The CDAI was calculated using six dietary antioxidants, based on two 24-hour dietary recall interviews, serving as comprehensive measure of the intake of these antioxidants. Weighted multivariable logistic regression and restricted cubic spline (RCS) analysis was conducted to investigate the association between CDAI and anemia. Furthermore, subgroup analyses were performed to enhance datas reliability.

Results: A total of 33914 participants were included in the study, among which 3,416 (10.07%) were diagnosed with anemia. The unadjusted model showed negative association between CDAI and anemia (odds ratio [OR]: 0.94; 95% confidence interval [95%CI]: 0.93-0.96; *P* < 0.001). After adjusting for all covariates , with each increase in CDAI level linked to 3% lower risk of anemia (OR: 0.97; 95%CI: 0.95 - 0.98; *P* < 0.001). Moreover, when CDAI was categorized into quartiles, the observed trend persisted (*P* < 0.001). The RCS analysis revealed linear negative relationship between CDAI and anemia (*P* for nonlinearity = 0.619). ~~Subgroup analysis of the antioxidant components of CDAI revealed that vitamins E, zinc, carotene, and selenium were independently associated with lower risk of anemia.~~ Except for sex, smoking, diabetes and hypertension, no statistically significant interactions were found in any subgroup analysis (*P* < 0.05 for interaction).

Conclusion: ~~An analysis of the NHANES database revealed correlation between higher CDAI levels and a reduced risk of anemia.~~ Our findings suggest that CDAI levels are inversely related to the prevalence of anemia. Consequently, monitoring individuals with low CDAI scores may facilitate the timely identification of anemia and enhance clinical decision-making. ~~Nevertheless, additional large-scale prospective studies are necessary to confirm the causal relationship between CDAI and anemia.~~

Keywords: Anemia; Antioxidant; Composite Dietary Antioxidant Index;

Cross-sectional study; Dietary. National Health and Nutrition Examination Survey.

Introduction

Anemia is a widespread global health concern that has been consistently linked to various negative outcomes in recent years [1]. It is characterized by an imbalance between the production and destruction of red blood cells (RBCs), resulting in inadequate oxygen delivery to vital tissues like the brain and heart [2]. The diagnosis of anemia typically relies on the hemoglobin level in the blood [3]. The prevalence and incidence of anemia have risen significantly, attributed to combination of increased nutrient deficiencies, chronic diseases, inherited hemoglobin disorders, and the use of specific medications [4, 5]. Anemia can have detrimental effects on cognitive and physical functions [6], leading to decreased economic productivity and higher morbidity and mortality rates [7], presenting significant health challenge in modern society. Early recognition of anemia presents an opportunity to delay or prevent the onset of the disease and enhance treatment outcomes. Consequently, identifying new indicators closely associated with anemia is of great significance for developing more effective anemia prevention strategies [4].

Oxidative stress is defined as an imbalance between the production of reactive oxygen species (ROS) and antioxidant defense mechanisms, and is acknowledged as a major factor in various conditions like inflammation, aging, cancer, and cardiovascular diseases [8]. The harmful effects of oxidative stress include the oxidation of cellular components, which can lead to cell death and organ damage through mechanisms [7] such as DNA damage, lipid peroxidation [8], and protein oxidation [9]. This damage further stimulates the production of ROS, worsening the underlying disease [10]. Numerous studies have emphasized a significant link between oxidative stress and the onset of anemia [9-11]. A modifiable risk factor for reducing oxidative stress is diet [12]. By including dietary antioxidants, individuals can alleviate or prevent oxidative stress-related diseases by neutralizing the harmful effects of ROS [13]. Consistent consumption of antioxidants can reduce oxidative stress levels and enhance the body's ability to withstand it [14]. Halima et al found that antioxidants exert protective effect against anemia and provide significant alternative benefits for RBC function. This is achieved by preventing lipid peroxidation in RBCs, increasing levels of reduced glutathione (GSH), and reducing RBC permeability [15]. As research enhances our understanding of nutrition and oxidative stress, there is an increasing interest in the role of antioxidant-rich diets in the prevention of anemia.

Recent research has highlighted the importance of consuming an antioxidant-rich diet to prevent anemia. The composite dietary antioxidant index (CDAI) was developed by Wright et al as a tool to assess antioxidant intake, serving as a composite score to evaluate dietary antioxidant consumption [16]. This index considers key nutrients such as vitamins A, vitamins C, and vitamins E, zinc, selenium, and carotenoids. While previous studies have mainly examined the

relationship between CDAI and different diseases, ~~like cancer [17], coronary heart disease [18], chronic kidney disease [19], and diabetes [20], showing a positive link between higher CDAI scores and reduced prevalence of these diseases,~~ Wang et al posited that the CDAI was positively correlated with lower prevalence of chronic kidney disease among American adults [17]. Similarly, Yu et al discovered that elevated CDAI scores were linked to decreased risk of colorectal cancer (CRC) and concluded that food-based antioxidants might contribute to lowering the risk of CRC in the general population [18]. Additionally, another study indicated that higher intake of dietary antioxidants, assessed through the Dietary Antioxidant Quality Score (DAQS) and CDAI, was associated with reduced risk of all-cause and cardiovascular disease mortality in adults with diabetes [19]. However, the specific connection between CDAI and anemia is yet to be conclusively determined.

A cross-sectional study was conducted using data from the National Health and Nutrition Examination Survey (NHANES) to assess the relationship between CDAI and anemia. The hypothesis posited that higher CDAI scores would correlate with decreased prevalence of anemia.

Materials and methods

NHANES database

The analysis is based on data from the NHANES, a cross-sectional survey conducted by the Centers for Disease Control (CDC) ~~and Prevention~~ and the National Center for Health Statistics (NCHS). The survey's objective is to evaluate the health and nutrition status of a representative population, encompassing institutionalized individuals [20]. Since 1999, NHANES has been an ongoing study that releases data every two years [21]. The database is available for free download from the official website. Approval for the study was obtained from the ~~National Center for Health Statistics~~ NCHS Research Ethics Review Board, and all participants provided written informed consent, and no external ethic approval was required for this study.

Study population

~~This study utilized NHANES data from 2003 to 2018, involving a total of 80,312 participants. After excluding 14,320 individuals without hemoglobin data and 4,656 without CDAI data, further removal of cases with missing covariate data resulted in final analysis sample of 34,439 participants. The exclusion criteria flow chart can be observed in Fig. 1.~~

This study utilized NHANES data spanning from 2003 to 2018, encompassing a total of 80,312 participants. After excluding individuals without hemoglobin data (N = 14,320), those lacking CDAI data (N = 4,656), and pregnant participants (N = 1,051), we further removed cases with missing covariate data. Ultimately, we obtained final sample size of 33914 participants. A flow chart depicting the exclusion criteria is presented in Fig 1.

Exposure and Outcomes

According to World Health Organization (WHO) guidelines, patients with anemia are defined as having hemoglobin (HB) levels below 12 g/dL for women and below 13 g/dL for men [22].

Data on dietary antioxidant intake were obtained from the average of two 24-hour dietary recall interviews conducted as part of the NHANES. The initial dietary recall was performed at mobile examination center (MEC) by trained interviewers who adhered to standardized protocol. During this face-to-face interview, detailed information regarding all food and beverages consumed by participants over the past 24 hours was collected. This was followed by second interview conducted via telephone 3 to 10 days later [23]. Utilizing average dietary intake data from two non-consecutive days is deemed more accurate than relying solely on data from a single day [24].~~The CDAI was developed using data from NHANES 24-hour dietary recall interviews conducted. The index utilized dietary data from two separate interviews to provide a more accurate representation of dietary intake [24].~~ To evaluate the overall exposure to dietary antioxidants, the intake of six key antioxidants (vitamin A, vitamin C, vitamin E, zinc, selenium, and carotene) was analyzed and quantified using to calculate the CDAI [25]. This involved standardizing the intake levels of the six antioxidants by subtracting the mean and dividing by the standard deviation, followed by summing up the standardized values:

$$\mathrm{CDAI} = \sum_{i=1}^{n} \left(\frac{x_n - \mu_n}{s_n} \right)$$

Covariates

Potential confounders considered in this study including age, sex, race~~/ethnicity~~, education levels, body mass index (BMI), poverty income ratio (PIR), smoking consumption, alcohol consumption, as well as comorbidities like hypertension, diabetes, cancer, and hyperlipidemia. Age was categorized into 40 years, ethnicity into non-Hispanic White, non-Hispanic Black, Mexican American, and others, and education levels into less than high school, high school, and more than high school. Marital status was divided into married/living with partner or widowed/divorced/separated/never married. PIR was categorized as low (□ 2.14) and high (□ 2.14). BMI was categorized as thin/normal (□18.5kg/m^2, 18.6 - 24.9kg/m^2), overweight (25.0 - 29.9 kg/m^2), and obese (□ 30 kg/m^2).

Smoking status was categorized into two groups: smokers, defined as those who had smoked at least 100 cigarettes in their lifetime (coded as 'yes'), and non-smokers (coded as 'no') [26]. Alcohol consumption was classified as 'yes' for those who had consumed at least 12 alcoholic drinks in the past year, and 'no' for others [27]. Participants were considered to have a history of diabetes, hypertension, and hyperlipidemia if they reported physicians diagnosis of these conditions. The

diagnosis of cancer required meeting two criteria: (1) positive response to 'ever been told they have cancer or a malignancy of any kind' (variable mcq220); (2) providing details for the inquiry 'what kind of cancer?' (variable mcq230A).

Statistical analysis

Participants were categorized into four groups based on quartiles CDAI. Continuous variables were presented as mean ± standard deviation (mean ± SD), while categorical variables were expressed as percentages. The associations between baseline characteristics and CDAI quartiles were evaluated using chi-square or t tests. Weighted logistic regression model was employed to investigate the relationship between CDAI and anemia, with results reported as adjusted odds ratio (OR) and 95% confidence intervals (CI). Model 1 had no adjustments, while model 2 included adjustments for gender, age, education level, and race. Model 3 further adjusted for PIR, BMI, smoking, drinking, hypertension, diabetes, and hyperlipidemia as covariates in addition to those in Model 2. CDAI was analyzed both as continuous and categorical variable to explore correlations. All statistical analyses were conducted using R 4.3.3 with appropriate weights, and statistical significance was defined as $P < 0.05$.

Ethics statement

The survey procedures are in accordance with the standards outlined in the declaration of Helsinki [28]. All information from the NHANES program is freely available to the public, therefore, the approval of the medical ethics committee board was not required [29].

Results

Baseline characteristics

This study involved 33914 patients who met strict inclusion and exclusion criteria, with 3416 (10.07%) of them diagnosed anemia. The average age of the participants was 47.35 ± 0.22 years. Patients were categorized into 4 groups based on CDAI quartiles, and their baseline characteristics are presented in table 1. Significant differences in age, sex, race, education levels, smoking consumption, alcohol consumption, PIR, BMI, diabetes, cancer, hypertension and hyperlipidemia were observed ($P < 0.001$). Higher GDAI levels were associated with certain characteristics such as younger age, non-Hispanic white married female, non-drinkers, non-smokers, higher BMI, higher PIR, higher education levels, absence of diabetes, cancer, hypertension, and anemia, but presence of hyperlipidemia. Univariable and multivariable logistic analyses showed significant link between CDAI and anemia (P <0.001) (Supplementary Table 1).

The association between CDAI and anemia

Table 2 presents the results of the weighted logistic regression analysis investigating the relationship between anemia and CDAI. The analysis demonstrated robust negative correlation between CDAI and anemia when considering CDAI as continuous variable in model 1 (OR: 0.94; 95%CI: 0.93 - 0.96), model 2 (OR: 0.96; 95%CI: 0.95 - 0.97) and model 3 (OR: 0.97; 95%CI: 0.95 - 0.98). Particularly noteworthy is the significant trend in model 3 (*P* for trend < 0.001) when CDAI was categorized into quartiles, indicating that higher CDAI scores were linked to lower likelihood of anemia. To further confirm this association, restricted cubic spline (RCS) analysis with 3 knots was performed, revealing linear negative correlation between CDAI and anemia (*P* for nonlinearity = 0.619), as depicted in Fig 2.

The association between antioxidant components and anemia

The relationship between the six antioxidant components of CDAI and anemia was further examined (Table 3), it was observed that vitamin A was linked to anemia in model 1 (OR: 1.00; 95%CI:1.00 - 1.00, *P* = 0.010). Vitamin C showed an association with anemia in models 1 and 2 (OR: 1.00; 95%CI: 1.00 - 1.00, *P* = 0.030 or *P* = 0.002). After adjusting for all variables, zinc (OR: 0.97; 95% CI: 0.97 - 0.98, *P* < 0.001), vitamin E (OR: 0.98; 95%CI: 0.98 - 0.99, *P* < 0.001), carotene (OR: 1.00; 95% CI: 1.00 - 1.00, *P* < 0.001), and selenium (OR: 1.00; 95% CI: 1.00 - 1.00, *P* = 0.010) were identified as independent factors associated with anemia.

Subgroup analysis

To further investigate the influence of CDAI on anemia risk within specific subgroups, stratified analysis was performed based on gender, age, race, PIR, BMI, hypertension, diabetes, and hyperlipidemia. Results indicated that except for sex, smoking consumption, and diabetes, there were no significant interactions between CDAI and anemia risk (*P* for interaction > 0.05, Fig 3). This indicates that the protective effect of CDAI on anemia is particularly pronounced in male non-smokers and non-diabetic individuals.

Discussion

This study is the first to investigate the relationship between CDAI and the ~~risk~~ prevalence of anemia based on data from NHANES. Upon adjusting for potential confounders, negative association between CDAI and anemia in American adults was observed, with linear trend established through dose-response analysis. Additionally, subgroup analysis indicated the protective effect of CDAI against anemia was

particularly significant among male nonsmokers and nondiabetic individuals. Clinically, these results suggest that anemia can be effectively prevented, diagnosed, and treated through appropriate dietary modifications rich in antioxidants.

~~There is growing evidence indicating that oxidative stress plays a significant role in the development and advancement of anemia. RBCs are particularly susceptible to oxidative damage when exposed to ROS from various sources [30]. Enika et al utilized mouse model, demonstrating that anemia can elevate oxidative stress in RBCs [31]. Similarly, Yoshihito et al established a connection between oxidative stress and autoimmune hemolytic anemia in mice, highlighting a correlation between the severity of anemia and intracellular ROS levels [32]. A recent study revealed that individuals with sickle cell anemia (SCA) experience disruptions in cellular redox balance and purine metabolite processing due to oxidative stress and changes in antioxidant markers [33].~~

Diet plays a crucial role in managing the body's oxidative stress levels [30], with dietary antioxidants being essential in lowering the risk of aging, cancer, diabetes, inflammation, liver disease, and cardiovascular disease. The fast-paced lifestyle of modern society leads to an increase in free radicals in the body, which can demage cells, tissues, and organs, ultimately impacting longevity. Antioxidants are key in fighting free radicals, thereby contributing to prevent both short-term and long-term diseases [31]. In this study, we focus on the CDAI for two primary reasons. Firstly, CDAI serves as a crucial indicator of dietary antioxidant capacity. Unlike single dietary antioxidant indicators, CDAI encompasses key antioxidant nutrients, including vitamin A, vitamin C, vitamin E, zinc, selenium, and carotene, thereby facilitating more comprehensive evaluation of overall diet quality. Secondly, research in related fields has garnered significant attention. A study within NHANES demonstrated negative linear correlation between CDAI and hypertension [32]. These results also propose that increasing CDAI levels through a diet rich in antioxidant nutrients could potentially reduce the occurrence of metabolic syndrome [12]. Similar, Ma et al in their study on the correlation between CDAI and coronary heart disease, they reported negative correlation, indicating that CDAI is inversely related to the incidence of coronary heart disease (Q4 vs Q1, OR: 0.65, 95%CI: 0.51 – 0.82, $P < 0.001$) [33]. While research on the relationship between CDAI and anemia is currently limited.

Our study reinforces these findings by showing an inverse relationship between

CDAI levels and anemia in American adults (OR: 0.97, 95%CI: 0.96 - 0.98). Specifically, higher CDAI scores were linked to lower prevalence of anemia. Our findings align with those of Zhang et al, who reported that among 5,880 participants, higher CDAI was associated with reduced likelihood of renal anemia (adjusted OR: 0.96, 95% CI: 0.94 - 0.98) [34]. In addition, studies focusing on specific anemic populations, such as patients with β - thalassemia, provide further insight into the relationship between anemia severity and antioxidant defenses. Allen et al suggest that the severity of anemia in these patients is linked to depletion of antioxidant defenses, indicating that antioxidant supplementation may be beneficial [35]. Andrea et al summarize the intricate interactions between antioxidant-rich foods and gut microbiota, inflammation, and obesity, positing that plant-based antioxidant foods, such as vegetables, fruits, and nuts, are essential for health maintenance [36]. Meanwhile, Jacques et al investigated the impact of various nutrients, including antioxidants like vitamin A and vitamin C, on iron metabolism to mitigate the risk of anemia [37]. This finding aligns with our results, which show that higher CDAI scores are associated with lower prevalence of anemia, thereby emphasizing the potential therapeutic role of antioxidants. Therefore, a balanced diet, such as the Mediterranean diet, along with specific foods like fish, fresh vegetables, and fruits, is recommended for individuals with low CDAI scores [36]. These foods are rich in essential components, including fiber, minerals, vitamins, and antioxidants, which can help prevent the occurrence of anemia.

~~While research on the relationship between CDAI and anemia is currently limited, studies in other areas have gained significant attention. A study within NHANES demonstrated a negative linear correlation between CDAI and hypertension [36]. These results also propose that increasing CDAI levels through a diet rich in antioxidant nutrients could potentially reduce the occurrence of metabolic syndrome [14]. Our study reinforces these findings by showing an inverse relationship between CDAI levels and anemia in American adults. Specifically, higher CDAI scores were linked to a lower prevalence of anemia, suggesting the need for further investigation to solidify the connection between antioxidant intake and anemia.~~

Subsequently, this study explored the correlation between antioxidant components and anemia, discovering that vitamins E, zinc, carotene, and selenium were independently linked to anemia after adjusting for all potential confounders.

Vitamin E is recognized as a crucial lipophilic antioxidant in biological membranes, capable of scavenging free radicals and acting as a chain-breaking antioxidant [38]. Severe vitamin E deficiency can lead to impaired immune response and hemolytic anemia caused by free radicals [39]. Sue et al highlighted that the lack of multiple trace element biomarkers (iron, zinc, selenium) was positively associated with anemia [40]. While Lisa et al showed that low zinc is an independent risk factor for anemia in school-age children and mediates the effect of low selenium on hemoglobin levels [41]. Carotene exert antioxidant effects through direct interactions with free radicals, which occur via electron or hydrogen atom transfer [42]. Additionally, carotene can be converted into vitamin A within the body, promoting iron absorption and metabolism, thereby exerting preventive effect against anemia [43]. However, these studies focused solely on specific antioxidants and did not examine potential synergies among various antioxidant nutrients. Given that our dietary intake typically includes multiple antioxidants, the CDAI seems to exert a comprehensive effect on individuals' pro-antioxidant status and underscores the advantages of thorough assessments of antioxidant exposure [17].

This study has several strengths. Firstly, it is the first cross-sectional survey to explore the relationship between CDAI and anemia, emphasizing the potential impact of dietary antioxidants in reducing anemia prevalence. Secondly, the findings indicate that the consumption of antioxidant- rich foods could potentially decrease the risk of anemia and offer dietary guidance to boost antioxidant intake for individuals with anemia.

However, this study also presents several limitations. Firstly, the dietary data in NHANES is based on self-reporting, which may introduce recall bias,and such errors are unavoidable. Secondly, the cross-sectional design complicates the establishment of causal relationship between CDAI and anemia, indicating that prospective multicenter studies will be necessary in the near future to validate our findings. Thirdly, the anemia data utilized in this study were derived from laboratory diagnoses and may not have accounted for the potential contributions of a history of anemia or biological differences, such as smoking or an increased incidence of thalassemia.

Lastly, although this study accounted for numerous clinical variables, potential unmeasured confounders, such as medication use and laboratory indicators, including liver and kidney function, iron levels, and vitamins, may still influence CDAI levels and their correlation with outcomes. Consequently, future research should aim to explore the specific associations between these variables more comprehensively.

Conclusion

~~In summary, A cross-sectional study utilizing data from 8 periods (2003-2018) of the NHANES database revealed negative association between CDAI and anemia in the American adults, even after accounting for potential confounders.~~ ~~This study presents a novel approach to exploring dietary intervention strategies aimed at reducing the prevalence of anemia.~~ This study revealed an inverse relationship between CDAI and the prevalence of anemia, with more pronounced effect observed in male nonsmokers and nondiabetic individuals. These findings offer valuable insights for healthcare providers to develop more targeted anemia screening and prevention strategies. Furthermore, Our study suggest the potential role of dietary modifications, particularly for individuals with lower CDAI scores, including the adoption of antioxidant-rich dietary patterns, such as iron supplements and plant-based diets, to help reduce the prevalence of anemia. Additionally, further large-scale prospective studies are necessary to confirm these findings.~~ and provide more precise and effective dietary recommendations for preventing anemia.~~

Reference

[1]. Lucca U, Tettamanti M, Tiraboschi P, Logroscino G, Landi C, Sacco L, et al. Incidence of dementia in the oldest-old and its relationship with age: The Monzino 80-plus population-based study. Alzheimer's & dementia : the journal of the Alzheimer's Association. 2020;16(3):472-81. http://dx.doi.org/10.1016/j.jalz.2019.09.083 PMID: 31786127.

[2]. Kassebaum NJ. The Global Burden of Anemia. Hematology/oncology clinics of North America. 2016;30(2):247-308. http://dx.doi.org/10.1016/j.hoc.2015.11.002 PMID: 27040955.

[3]. Bansal PG, Toteja GS, Bhatia N, Gupta S, Kaur M, Adhikari T, et al. Comparison of haemoglobin estimates using direct & indirect cyanmethaemoglobin methods. The Indian journal of medical research. 2016;144(4):566-71. http://dx.doi.org/10.4103/0971-5916.200882 PMID: 28256465.

[4]. Chen S, Xiao J, Cai W, Lu X, Liu C, Dong Y, et al. Association of the systemic immune-inflammation index with anemia: a population-based study. Frontiers in immunology. 2024;15:1391573. http://dx.doi.org/10.3389/fimmu.2024.1391573 PMID: 38799419.
[5]. Stevens GA, Paciorek CJ, Flores-Urrutia MC, Borghi E, Namaste S, Wirth JP, et al. National, regional, and global estimates of anaemia by severity in women and children for 2000-19: a pooled analysis of population-representative data. The Lancet Global health. 2022;10(5):e627-e39. http://dx.doi.org/10.1016/s2214-109x(22)00084-5 PMID: 35427520.
[6]. Kinyoki D, Osgood-Zimmerman AE, Bhattacharjee NV, Kassebaum NJ, Hay SI. Anemia prevalence in women of reproductive age in low- and middle-income countries between 2000 and 2018. Nature medicine. 2021;27(10):1761-82. http://dx.doi.org/10.1038/s41591-021-01498-0 PMID: 34642490.
[7]. Martinsson A, Andersson C, Andell P, Koul S, Engström G, Smith JG. Anemia in the general population: prevalence, clinical correlates and prognostic impact. European journal of epidemiology. 2014;29(7):489-98. http://dx.doi.org/10.1007/s10654-014-9929-9 PMID: 24952166.
[8]. Du W, Adam Z, Rani R, Zhang X, Pang Q. Oxidative stress in Fanconi anemia hematopoiesis and disease progression. Antioxidants & redox signaling. 2008;10(11):1909-21. http://dx.doi.org/10.1089/ars.2008.2129 PMID: 18627348.
[9]. Meroño T, Dauteuille C, Tetzlaff W, Martín M, Botta E, Lhomme M, et al. Oxidative stress, HDL functionality and effects of intravenous iron administration in women with iron deficiency anemia. Clinical nutrition (Edinburgh, Scotland). 2017;36(2):552-8. http://dx.doi.org/10.1016/j.clnu.2016.02.003 PMID: 26926576.
[10]. Castilhos LG, de Oliveira JS, Adefegha SA, Magni LP, Doleski PH, Abdalla FH, et al. Increased oxidative stress alters nucleosides metabolite levels in sickle cell anemia. Redox report : communications in free radical research. 2017;22(6):451-9. http://dx.doi.org/10.1080/13510002.2017.1288973 PMID: 28209096.
[11]. Podkowińska A, Formanowicz D. Chronic Kidney Disease as Oxidative Stress- and Inflammatory-Mediated Cardiovascular Disease. Antioxidants (Basel, Switzerland). 2020;9(8). http://dx.doi.org/10.3390/antiox9080752 PMID: 32823917.
[12]. Zhu Y, Bing J, Zheng L, Hao P, Teng X, Wan L. The association between composite dietary antioxidant index and the metabolic syndrome: NHANES 2007–2018. Journal of Functional Foods. 2024;116:106200. http://dx.doi.org/https://doi.org/10.1016/j.jff.2024.106200 PMID:
[13]. Corrochano AR, Buckin V, Kelly PM, Giblin L. Invited review: Whey proteins as antioxidants and promoters of cellular antioxidant pathways. Journal of dairy science. 2018;101(6):4747-61. http://dx.doi.org/10.3168/jds.2017-13618 PMID: 29605324.
[14]. Ogura Y, Kitada M, Koya D. Sirtuins and Renal Oxidative Stress. Antioxidants (Basel, Switzerland). 2021;10(8). http://dx.doi.org/10.3390/antiox10081198 PMID: 34439446.
[15]. Al Balushi H, Hannemann A, Rees D, Brewin J, Gibson JS. The Effect of Antioxidants on the Properties of Red Blood Cells From Patients With Sickle Cell Anemia. Frontiers in physiology. 2019;10:976. http://dx.doi.org/10.3389/fphys.2019.00976 PMID: 31456691.
[16]. Wright ME, Mayne ST, Stolzenberg-Solomon RZ, Li Z, Pietinen P, Taylor PR, et al. Development of a comprehensive dietary antioxidant index and application to lung cancer risk in a cohort of male smokers. American journal of epidemiology. 2004;160(1):68-76. http://dx.doi.org/10.1093/aje/kwh173 PMID: 15229119.

[17]. Wang M, Huang ZH, Zhu YH, He P, Fan QL. Association between the composite dietary antioxidant index and chronic kidney disease: evidence from NHANES 2011-2018. Food & function. 2023;14(20):9279-86. http://dx.doi.org/10.1039/d3fo01157g PMID: 37772927.
[18]. Yu YC, Paragomi P, Wang R, Jin A, Schoen RE, Sheng LT, et al. Composite dietary antioxidant index and the risk of colorectal cancer: Findings from the Singapore Chinese Health Study. International journal of cancer. 2022;150(10):1599-608. http://dx.doi.org/10.1002/ijc.33925 PMID: 35001362.
[19]. Wang W, Wang X, Cao S, Duan Y, Xu C, Gan D, et al. Dietary Antioxidant Indices in Relation to All-Cause and Cause-Specific Mortality Among Adults With Diabetes: A Prospective Cohort Study. Front Nutr. 2022;9:849727. http://dx.doi.org/10.3389/fnut.2022.849727 PMID: 35600816.
[20]. Qing L, Zhu Y, Yu C, Zhang Y, Ni J. Exploring the association between dietary Inflammatory Index and chronic pain in US adults using NHANES 1999-2004. Scientific reports. 2024;14(1):8726. http://dx.doi.org/10.1038/s41598-024-58030-w PMID: 38622145.
[21]. Scinicariello F, Buser MC, Feroe AG, Attanasio R. Antimony and sleep-related disorders: NHANES 2005-2008. Environmental research. 2017;156:247-52. http://dx.doi.org/10.1016/j.envres.2017.03.036 PMID: 28363141.
[22]. Safiri S, Kolahi AA, Noori M, Nejadghaderi SA, Karamzad N, Bragazzi NL, et al. Burden of anemia and its underlying causes in 204 countries and territories, 1990-2019: results from the Global Burden of Disease Study 2019. Journal of hematology & oncology. 2021;14(1):185. http://dx.doi.org/10.1186/s13045-021-01202-2 PMID: 34736513.
[23]. Pannucci TE, Thompson FE, Bailey RL, Dodd KW, Potischman N, Kirkpatrick SI, et al. Comparing Reported Dietary Supplement Intakes between Two 24-Hour Recall Methods: The Automated Self-Administered 24-Hour Dietary Assessment Tool and the Interview-Administered Automated Multiple Pass Method. Journal of the Academy of Nutrition and Dietetics. 2018;118(6):1080-6. http://dx.doi.org/10.1016/j.jand.2018.02.013 PMID: 29803270.
[24]. Yang T, Yi J, He Y, Zhang J, Li X, Ke S, et al. Associations of Dietary Fats with All-Cause Mortality and Cardiovascular Disease Mortality among Patients with Cardiometabolic Disease. Nutrients. 2022;14(17). http://dx.doi.org/10.3390/nu14173608 PMID: 36079863.
[25]. Xu Q, Qian X, Sun F, Liu H, Dou Z, Zhang J. Independent and joint associations of dietary antioxidant intake with risk of post-stroke depression and all-cause mortality. Journal of affective disorders. 2023;322:84-90. http://dx.doi.org/10.1016/j.jad.2022.11.013 PMID: 36372128.
[26]. Wang MY, Li J, Peng HY, Liu J, Huang KL, Li L, et al. Patients with different types of arthritis may be at risk for major depression: results from the National Health and Nutrition Examination Survey 2007-2018. Annals of palliative medicine. 2021;10(5):5280-8. http://dx.doi.org/10.21037/apm-21-279 PMID: 33977741.
[27]. King DE, Xiang J. The Dietary Inflammatory Index Is Associated With Diabetes Severity. Journal of the American Board of Family Medicine : JABFM. 2019;32(6):801-6. http://dx.doi.org/10.3122/jabfm.2019.06.190092 PMID: 31704748.
[28]. Xu H, Wen Q, Xing X, Chen Y, Zhu Q, Tan M, et al. High Dietary Inflammatory Index increases the risk of female infertility: An analysis of NHANES 2013-2018. Nutrition

research (New York, NY). 2024;125:50-60. http://dx.doi.org/10.1016/j.nutres.2024.02.006 PMID: 38503022.

[29]. Luo J, Chen T, Chen Y, Huang ZM, Li XJ, Chen HK, et al. The association between homocysteine and bacterial vaginosis: results from NHANES 2001-2004. Scientific reports. 2023;13(1):21388. http://dx.doi.org/10.1038/s41598-023-45494-5 PMID: 38049434.

[30]. Teng TQ, Liu J, Hu FF, Li QQ, Hu ZZ, Shi Y. Association of composite dietary antioxidant index with prevalence of stroke: insights from NHANES 1999-2018. Frontiers in immunology. 2024;15:1306059. http://dx.doi.org/10.3389/fimmu.2024.1306059 PMID: 38524123.

[31]. Neha K, Haider MR, Pathak A, Yar MS. Medicinal prospects of antioxidants: A review. European journal of medicinal chemistry. 2019;178:687-704. http://dx.doi.org/10.1016/j.ejmech.2019.06.010 PMID: 31228811.

[32]. Wu M, Si J, Liu Y, Kang L, Xu B. Association between composite dietary antioxidant index and hypertension: insights from NHANES. Clinical and experimental hypertension (New York, NY : 1993). 2023;45(1):2233712. http://dx.doi.org/10.1080/10641963.2023.2233712 PMID: 37439538.

[33]. Ma R, Zhou X, Zhang G, Wu H, Lu Y, Liu F, et al. Association between composite dietary antioxidant index and coronary heart disease among US adults: a cross-sectional analysis. BMC public health. 2023;23(1):2426. http://dx.doi.org/10.1186/s12889-023-17373-1 PMID: 38053099.

[34]. Zhang L, Yin D, Zhu T, Geng L, Gan L, Ou S, et al. Composite dietary antioxidant index is associated with renal anemia: a cross-sectional study. International urology and nephrology. 2024. http://dx.doi.org/10.1007/s11255-024-04157-8 PMID: 39044023.

[35]. Allen A, Perera S, Mettananda S, Rodrigo R, Perera L, Darshana T, et al. Oxidative status in the β-thalassemia syndromes in Sri Lanka; a cross-sectional survey. Free radical biology & medicine. 2021;166:337-47. http://dx.doi.org/10.1016/j.freeradbiomed.2021.02.028 PMID: 33677065.

[36]. Deledda A, Annunziata G, Tenore GC, Palmas V, Manzin A, Velluzzi F. Diet-Derived Antioxidants and Their Role in Inflammation, Obesity and Gut Microbiota Modulation. Antioxidants (Basel, Switzerland). 2021;10(5). http://dx.doi.org/10.3390/antiox10050708 PMID: 33946864.

[37]. Berger J, Wieringa FT, Lacroux A, Dijkhuizen MA. Strategies to prevent iron deficiency and improve reproductive health. Nutrition reviews. 2011;69 Suppl 1:S78-86. http://dx.doi.org/10.1111/j.1753-4887.2011.00436.x PMID: 22043887.

[38]. Takahashi M, Tsuchiya J, Niki EJAotNYAoS. Mode of Action of Vitamin E as Antioxidant in the Membranes as Studied by Spin Labeling. 1989;570. PMID:

[39]. Jilani T, Iqbal MP. Vitamin E deficiency in South Asian population and the therapeutic use of alpha-tocopherol (Vitamin E) for correction of anemia. Pakistan journal of medical sciences. 2018;34(6):1571-5. http://dx.doi.org/10.12669/pjms.346.15880 PMID: 30559825.

[40]. MacDonell SO, Miller JC, Harper MJ, Reid MR, Haszard JJ, Gibson RS, et al. Multiple Micronutrients, Including Zinc, Selenium and Iron, Are Positively Associated with Anemia in New Zealand Aged Care Residents. Nutrients. 2021;13(4). http://dx.doi.org/10.3390/nu13041072 PMID: 33806205.

[41]. Houghton LA, Parnell WR, Thomson CD, Green TJ, Gibson RS. Serum Zinc Is a Major Predictor of Anemia and Mediates the Effect of Selenium on Hemoglobin in School-Aged Children in a Nationally Representative Survey in New Zealand. The Journal of nutrition. 2016;146(9):1670-6. http://dx.doi.org/10.3945/jn.116.235127 PMID: 27466609.
[42]. Zhao M, Zhang D, Zhang Q, Lin Y, Cao H. Association between composite dietary antioxidant index and hyperlipidemia: a cross-sectional study from NHANES (2005-2020). Scientific reports. 2024;14(1):15935. http://dx.doi.org/10.1038/s41598-024-66922-0 PMID: 38987566.
[43]. Cañete A, Cano E, Muñoz-Chápuli R, Carmona R. Role of Vitamin A/Retinoic Acid in Regulation of Embryonic and Adult Hematopoiesis. Nutrients. 2017;9(2). http://dx.doi.org/10.3390/nu9020159 PMID: 28230720.

Table 1. The weighted baseline characteristics by CDAI quartiles.

Characteristics	Total (N = 33914)	Quartiles of CDAI				*P* value
		Q1 (N = 8482)	Q2 (N = 8477)	Q3 (N = 8477)	Q4 (N = 8478)	
**Age (years)**	47.35 ± 0.22	47.35 ± 0.22	47.84 ± 0.30	47.99 ± 0.26	47.66 ± 0.32	< **0.001**
**Sex**						< **0.001**
Female	17110 (50.45)	4622 (54.49)	4182 (49.33)	4175 (49.25)	4131 (48.73)	
Male	16804 (49.55)	3860 (45.51)	4295 (50.67)	4302 (50.75)	4347 (51.27)	
**Race**						< **0.001**
Non-Hispanic black	6977 (20.57)	2124 (25.04)	1712 (20.20)	1546 (18.24)	1595 (18.81)	
Non-Hispanic white	15510 (45.73)	3555 (41.91)	3921 (46.25)	4070 (48.01)	3964 (46.76)	
Mexican American	5323 (15.70)	1287 (15.17)	1368 (16.14)	1352 (15.95)	1316 (15.52)	
Other Hispanic	6104 (18.00)	2124 (25.04)	1476 (17.41)	1509 (17.80)	1603 (18.91)	
**Education levels**						< **0.001**
High school	4704 (13.87)	1471 (17.34)	1207 (14.24)	1067 (12.59)	959 (11.31)	
Less than high school	3467 (10.22)	1221 (14.40)	909 (10.72)	748 (8.82)	589 (6.95)	
More than high school	25743 (75.91)	5790 (68.26)	6361 (75.04)	6662 (78.59)	6930 (81.74)	
**Marital status**						< **0.001**
Divorced	3763 (11.10)	1098 (12.95)	899 (10.61)	889 (10.61)	877 (10.34)	
Living with partner	2660 (7.84)	677 (7.98)	611 (7.21)	642 (7.57)	730 (8.61)	
Married	17708 (52.21)	3971 (46.82)	4541 (53.57)	4653 (54.89)	4543 (53.59)	
Never married	5905 (17.42)	1549 (18.26)	1416 (16.70)	1378 (16.26)	1562 (18.42)	
Separated/Windowed	3878 (11.43)	1187 (13.99)	1010 (11.91)	915 (10.79)	766 (9.04)	

**Drinking consumption**						**< 0.001**
No	24345 (71.78)	6494 (72.88)	6184 (72.95)	5874 (69.29)	5793 (68.37)	
Yes	9569 (28.22)	1988 (27.12)	2293 (27.05)	2603 (30.71)	2685 (33.83)	
**Smoking consumption**						**< 0.001**
No	18318 (54.01)	4204 (49.56)	4537 (53.52)	4716 (55.63)	4861 (57.34)	
Yes	15596 (45.99)	4278 (50.44)	3940 (46.48)	3761 (44.37)	3617 (42.66)	
**BMI**						**< 0.001**
Normal	9255 (27.29)	2249 (26.51)	2218 (26.17)	2263 (26.70)	2525 (29.78)	
Thin	518 (1.53)	156 (1.84)	110 (1.30)	113 (1.33)	139 (1.64)	
Obese	12865 (37.93)	3312 (39.05)	3247 (38.30)	3229 (38.09)	3077 (36.30)	
Overweight	11276 (33.25)	2765 (32.60)	2902 (34.23)	2872 (33.88)	2737 (32.28)	
**PIR**						**< 0.001**
≥2.14	16988 (50.09)	3426 (40.39)	4192 (49.45)	4643 (54.77)	4727 (55.76)	
<2.14	16926 (49.91)	5056 (59.61)	4285 (50.55)	3834 (45.23)	3751 (44.24)	
**Hypertension**						**< 0.001**
No	19345 (57.04)	4463 (52.62)	4711 (55.57)	4972 (58.65)	5199 (61.32)	
Yes	14569 (42.96)	4019 (47.38)	3766 (44.43)	3505 (41.35)	3279 (38.68)	
**Hyperlipidemia**						**< 0.001**
No	9725 (28.68)	2237 (26.37)	2331 (27.50)	2443 (28.82)	2714 (32.01)	
Yes	24189 (71.32)	6245 (73.62)	6146 (72.50)	6034 (71.18)	5764 (67.99)	
**Diabetes**						**< 0.001**
Borderline	746 (2.20)	181 (2.13)	192 (2.26)	179 (2.11)	194 (2.29)	
No	28883 (85.17)	7051 (83.13)	7112 (83.90)	7306 (86.19)	7414 (87.45)	
Yes	4285 (12.63)	1250 (14.74)	1173 (13.84)	992 (11.70)	870 (10.26)	
**Anemia**						**< 0.001**
No	30498 (89.93)	7440 (87.72)	7589 (89.52)	7655 (90.30)	7814 (92.17)	
Yes	3416 (10.07)	1042 (12.28)	888 (10.48)	822 (9.70)	664 (7.83)	
**Cancer**						**0.030**
No	30678 (90.46)	7681 (90.56)	7590 (89.54)	7662 (90.39)	7745 (91.35)	
Yes	3236 (9.54)	801 (9.44)	887 (10.46)	815 (9.61)	733 (8.65)	

The first quartile of CDAI is defined as Q1, with the subsequent quartiles being defined as Q2, Q3, and Q4. Note: Data are presented as mean (SD) or n (%). BMI, body mass index; HB, hemoglobin; PIR, poverty income ratio; CDAI, composite dietary antioxidant indexes. *P* values < 0.05 are in bold.

Table 2. The association between CDAI and anemia.

Exposure	Model 1	Model 2	Model 3

	OR [95%CI]	*P*	OR [95%CI]	*P*	OR [95%CI]	*P*
CDAI	0.94 (0.93,0.96)	**<0.001**	0.96 (0.95,0.97)	**<0.001**	0.97 (0.95,0.98)	**<0.001**
CDAIQ						
Q1	ref		ref		ref	
Q2	0.85(0.75,0.97)	**0.010**	0.98 (0.86,1.11)	0.690	0.99 (0.86,1.13)	0.870
Q3	0.74 (0.65,0.84)	**<0.001**	0.89 (0.78,1.02)	0.140	0.93 (0.81,1.07)	0.490
Q4	0.56 (0.49,0.64)	**<0.001**	0.68 (0.59,0.77)	**<0.001**	0.71 (0.62,0.81)	**<0.001**
P for trend		**<0.001**		**<0.001**		**<0.001**

Model 1: no covariates were adjusted.
Model 2: adjusted for gender, age, education level and race.
Model 3: adjusted for gender, age, race, education level, PIR, BMI, smoke, drink, hypertension, diabetes and hyperlipidemia. *P* values < 0.05 are in bold.

Table 3. The weighted logistic regression analysis of the association between antioxidant components and anemia.

antioxidant components	Model 1		Model 2		Model 3	
	OR [95%CI]	*P*	OR [95%CI]	*P*	OR [95%CI]	*P*
Vitamins A (mg)	1.00 (1.00,1.00)	**0.010**	1.00 (1.00,1.00)	0.890	1.00 (1.00,1.00)	0.910
Vitamins C (mg)	1.00 (1.00,1.00)	**0.030**	1.00 (1.00,1.00)	**0.020**	1.00 (1.00,1.00)	0.050
Vitamins E (mg)	0.96 (0.95,0.97)	**<0.001**	0.98 (0.97,0.99)	**< 0.001**	0.98 (0.98,0.99)	**<0.001**
Zinc (mg)	0.94 (0.94,0.95)	**<0.001**	0.97 (0.96,0.98)	**<0.001**	0.97 (0.97,0.98)	**<0.001**
Selenium (mg)	0.99 (0.99,1.00)	**<0.001**	1.00 (1.00,1.00)	**<0.001**	1.00 (1.00,1.00)	**<0.001**
Carotene (mg)	1.00 (1.00,1.00)	**<0.001**	1.00 (1.00,1.00)	**0.001**	1.00 (1.00,1.00)	**0.010**

Model 1: no covariates were adjusted.
Model 2: adjusted for gender, age, education level and race.
Model 3: adjusted for gender, age, race, education level, PIR, BMI, smoke, drink, hypertension, diabetes and hyperlipidemia. *P* values < 0.05 are in bold.

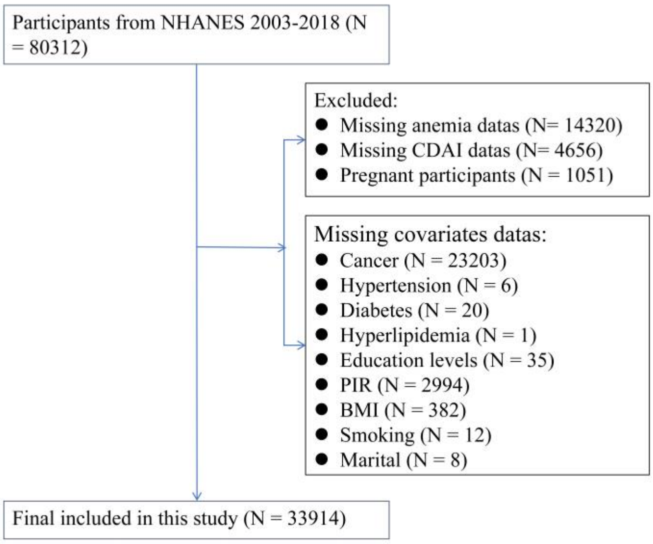

Fig 1. **Flow chart of participant selection in NHANES.** Abbreviations: NHANES, National Health and Nutrition Examination Survey; CDAI, Composite Dietary Antioxidant Index

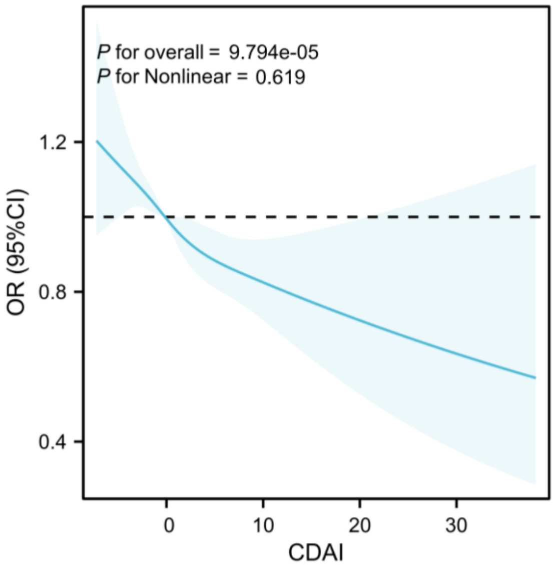

Fig 2. The RCS curve of the association between CDAI and anemia. RCS regression was adjusted by model 3. RCS, restricted cubic spline; CDAI, composite dietary antioxidant index; OR, odds ratio.

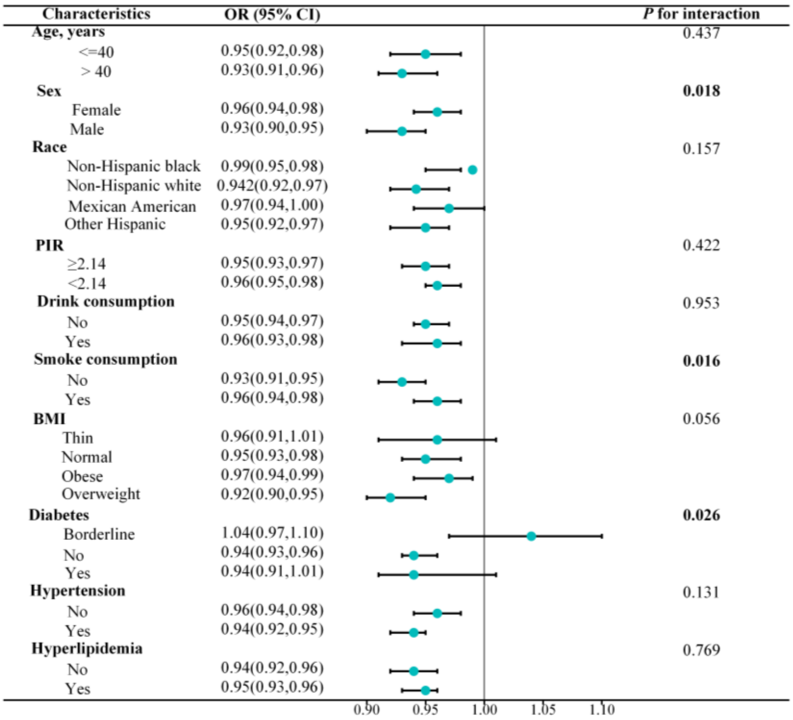

Fig 3. **Subgroups analyses for the association between CDAI and anemia.** BMI, body mass index; PIR, poverty income ratio.

Cover Letter

Dear Editors and Reviewers,

Thank you for giving us an opportunity to revise our manuscript. On behalf of all the contributing authors, I would like to express our sincere appreciations of your letter and reviewers' constructive comments concerning our article entitled **"Association between composite dietary antioxidant indices and anemia: NHANES 2003–2018" (Number: PONE-D-24-28259)**. Those comments are all valuable and very helpful for revising and improving our paper. We have studied comments carefully and have made extensive modifications which we hope meet with approval. The editors and reviewers' comments are laid out below in blue font and specific concerns have been numbered. Our responses are provided in normal font, and the specific locations of changes and additions to the manuscript are highlighted in red text. Additionally, we have included grant numbers and funding sources in the funding information section. We have also uploaded the proof of polishing the manuscript. If there are any specific location problems, we will continue to modify.

Thank you and best regards.

Yours sincerely,

Xiaoxia Gou, MD
Department of Head and Neck Oncology
The Second Affiliated Hospital of Zunyi Medical University
Zunyi, Guizhou 563000,China.
E-mail: gouxx2020@163.com

Response to comments from Editors and Reviewers

Editorial comment

1. ***Comment***: Please ensure that your manuscript meets PLOS ONE's style requirements, including those for file naming. The PLOS ONE style templates can be found at https://journals.plos.org/plosone/s/file?id=wjVg/PLOSOne_formatting_sample_main_body.pdf and https://journals.plos.org/plosone/s/file?id=ba62/PLOSOne_formatting_sample_title_authors_affiliations.pdf.

1. Response: Thank you for your kind reminder. In response to your suggestions, we revisited the PLOS ONE submission guidelines and implemented several changes, including partial rearrangements of text fonts, figure captions, references, and other elements within the manuscript.

2. ***Comment***: Please ensure that you include a title page within your main document. Wedo appreciate that you have a title page document uploaded as a separate file,however, as per our author guidelines (http://journals.plos.org/plosone/s/submission-guidelines#loc-title-page) we do require this to be part of the manuscript file itself and not uploaded separately. Could you therefore please include the title page into the beginning of your manuscript file itself, listing all authors and affiliations.

2. Response: Thanks for your reminding. We will ensure that title page is included at the beginning of the manuscript.

3. ***Comment***: We note that the grant information you provided in the 'Funding Information' and 'Financial Disclosure' sections do not match. When you resubmit, please ensure that you provide the correct grant numbers for the awards you received for your study in the 'Funding Information' section.

3. Response: We sincerely thank you for your valuable reminding and have added the grant number to the grant information section.

This study was supported by the National Natural Science Foundation of China (82460465); The Natural Science Foundation of Guizhou Province (Qian Ke He Basic Project ZK [2024]346); The Guizhou Anti-Cancer Association Science and Technology Plan Project (Anti-Cancer Association Science and Technology 006[2023]); The Natural Science Foundation of Guizhou Province (Qian Ke He Basic Project ZK [2023]525).

4. *Comment*: Thank you for stating the following financial disclosure: “1.Guizhou Provincial Department of Science and Technology project 2.Science and Technology Program of Guizhou Anti-Cancer Association” Please state what role the funders took in the study. If the funders had no role, please state: "The funders had no role in study design, data collection and analysis, decision to publish, or preparation of the manuscript." If this statement is not correct you must amend it as needed. Please include this amended Role of Funder statement in your cover letter; we will change the online submission form on your behalf.

4. Response: Thank you for your kind reminder. The funder participated in this study, and the specific funding information has been matched and updated in the cover letter. Eg: **Xiaoxia Gou**: The National Natural Science Foundation of China (82460465); The Natural Science Foundation of Guizhou Province (Qian Ke He Basic Project ZK [2024]346); The Guizhou Anti-Cancer Association Science and Technology Plan Project (Anti-Cancer Association Science and Technology 006[2023]);

Gang Huang: The Natural Science Foundation of Guizhou Province (Qian Ke He Basic Project ZK [2023]525).

5. *Comment*: Please include a separate caption for each figure in your manuscript.

5. Response: Thanks for your reminding. Each figure in our manuscript is accompanied by distinct figure caption. Please refer upload Graphical Abstract file. Additionally, we have re-uploaded the revised figure, Please refer upload other files (rev.Figure) .

6. *Comment*: Please include your tables as part of your main manuscript and remove the individual files. Please note that supplementary tables (should remain/ be uploaded) as separate "supporting information" files

6. Response: Thank you once again for your kind reminder. We have included the tables as part of the manuscript and ensured that the supplementary tables are uploaded as separate 'Supporting Information' files. Please refer upload other files (table).

Review Comments

Reviewer #1:

1.*Comment*: This manuscript provides a background on the topic, however, it need a clearer or specific research gab. Additionally, the authors should provide more details on the rationale for selecting the Composite Dietary Antioxidant Index (CDAI). About The CDAI, how to use CDAI, and if you compare it with another method, what kind of comparative advantage that you get.

1. Response: Thank you immensely for your inquiry, your insights are profoundly valuable. Indeed, our introduction section had overlooked critical aspects, particularly a detailed exploration of the CDAI and focus on comparative research. To clearly articulate the research gap and align with your recommendations, we have enriched our introduction section with the following revised content.

1) Based on your recommendations, we have added a section in the Introduction section about specific research gab, with the specifics outlined below:

① (Manuscript, Introduction section, page 3, line 69-72): The prevalence and incidence of anemia have risen significantly, attributed to combination of increased nutrient deficiencies, chronic diseases, inherited hemoglobin disorders, and the use of specific medications [4,5].

Reference:

[4]. Chen S, Xiao J, Cai W, Lu X, Liu C, Dong Y, et al. Association of the systemic immune-inflammation index with anemia: a population-based study. Frontiers in immunology. 2024;15:1391573. http://dx.doi.org/10.3389/fimmu.2024.1391573 PMID: 38799419.

[5]. Stevens GA, Paciorek CJ, Flores-Urrutia MC, Borghi E, Namaste S, Wirth JP, et al. National, regional, and global estimates of anaemia by severity in women and children for 2000-19: a pooled analysis of population-representative data. The Lancet Global health. 2022;10(5):e627-e39. http://dx.doi.org/10.1016/s2214-109x(22)00084-5 PMID: 35427520.

② (Manuscript, Introduction section, page 3, line 75-78): Early recognition of anemia presents an opportunity to delay or prevent the onset of the disease and enhance treatment outcomes. Consequently, identifying new indicators closely associated with anemia is of great significance for developing more effective anemia prevention strategies [4].

Reference:

[4]. Chen S, Xiao J, Cai W, Lu X, Liu C, Dong Y, et al. Association of the systemic immune-inflammation index with anemia: a population-based study. Frontiers in immunology. 2024;15:1391573. http://dx.doi.org/10.3389/fimmu.2024.1391573 PMID: 38799419.

③(Manuscript, Introduction section, page 4, line 91-93): As research enhances our understanding of nutrition and oxidative stress, there is an increasing interest in the role of antioxidant-rich diets in the prevention of anemia.

2) We revised the paper and added necessary descriptions about more details for the CDAI in the introduction section and discussion section. The details are as follows :

① (Manuscript, Introduction section, page 4, line 94-96): The composite dietary antioxidant index (CDAI) was developed by Wright et al as a tool to assess antioxidant intake, serving as a composite score to evaluate dietary antioxidant consumption [16].

Reference:

[16] Wright ME, Mayne ST, Stolzenberg-Solomon RZ, Li Z, Pietinen P, Taylor PR, et al. Development of a comprehensive dietary antioxidant index and application to lung cancer risk in a

cohort of male smokers. American journal of epidemiology. 2004;160(1):68-76. http://dx.doi.org/10.1093/aje/kwh173 PMID: 15229119.

② (Manuscript, Introduction section, page 4, line 98-106): Wang et al posited that the CDAI was positively correlated with lower prevalence of chronic kidney disease among American adults [17]. Similarly, Yu et al discovered that elevated CDAI scores were linked to decreased risk of colorectal cancer (CRC) and concluded that food-based antioxidants might contribute to lowering the risk of CRC in the general population [18]. Additionally, another study indicated that higher intake of dietary antioxidants, assessed through the Dietary Antioxidant Quality Score (DAQS) and CDAI, was associated with reduced risk of all-cause and cardiovascular disease mortality in adults with diabetes [19].

Reference:

[17]. Wang M, Huang ZH, Zhu YH, He P, Fan QL. Association between the composite dietary antioxidant index and chronic kidney disease: evidence from NHANES 2011-2018. Food & function. 2023;14(20):9279-86. http://dx.doi.org/10.1039/d3fo01157g PMID: 37772927.

[18]. Yu YC, Paragomi P, Wang R, Jin A, Schoen RE, Sheng LT, et al. Composite dietary antioxidant index and the risk of colorectal cancer: Findings from the Singapore Chinese Health Study. International journal of cancer. 2022;150(10):1599-608. http://dx.doi.org/10.1002/ijc.33925 PMID: 35001362.

[19]. Wang W, Wang X, Cao S, Duan Y, Xu C, Gan D, et al. Dietary Antioxidant Indices in Relation to All-Cause and Cause-Specific Mortality Among Adults With Diabetes: A Prospective Cohort Study. Front Nutr. 2022;9:849727. http://dx.doi.org/10.3389/fnut.2022.849727 PMID: 35600816.

③ (Manuscript, Discussion section on Page 9, line 251-256): In this study, we focus on the CDAI for two primary reasons. Firstly, CDAI serves as a crucial indicator of dietary antioxidant capacity. Unlike single dietary antioxidant indicators, CDAI encompasses key antioxidant nutrients, including vitamin A, vitamin C, vitamin E, zinc, selenium, and carotene, thereby facilitating more comprehensive evaluation of overall diet quality. Secondly, research in related fields has garnered significant attention.

④ (Manuscript, Discussion section on Page 9, line 259-262): Similar, Ma et al in

their study on the correlation between CDAI and coronary heart disease, they reported negative correlation, indicating that CDAI is inversely related to the incidence of coronary heart disease (Q4 vs Q1, OR: 0.65, 95%CI: 0.51 - 0.82, $P < 0.001$) [33].

Reference:

[33]. Ma R, Zhou X, Zhang G, Wu H, Lu Y, Liu F, et al. Association between composite dietary antioxidant index and coronary heart disease among US adults: a cross-sectional analysis. BMC public health. 2023;23(1):2426. http://dx.doi.org/10.1186/s12889-023-17373-1 PMID: 38053099.

⑤ (Manuscript, Discussion section, page 10, Line 264-269) : Our study reinforces these findings by showing an inverse relationship between CDAI levels and anemia in American adults (OR: 0.97, 95%CI: 0.96 - 0.98). Specifically, higher CDAI scores were linked to lower prevalence of anemia. Our findings align with those of Zhang et al, who reported that among 5,880 participants, higher CDAI was associated with reduced likelihood of renal anemia (adjusted OR: 0.96, 95% CI: 0.94 - 0.98) [34].

Reference:

[34]. Zhang L, Yin D, Zhu T, Geng L, Gan L, Ou S, et al. Composite dietary antioxidant index is associated with renal anemia: a cross-sectional study. International urology and nephrology. 2024. http://dx.doi.org/10.1007/s11255-024-04157-8 PMID: 39044023.

⑥ (Manuscript, Discussion section on Page 10, line 279-281): This finding aligns with our results, which show that higher CDAI scores are associated with lower prevalence of anemia, thereby emphasizing the potential therapeutic role of antioxidants.

⑦ (Manuscript, Discussion section on Page 11, line 299-304): However, these studies focused solely on specific antioxidants and did not examine potential synergies among various antioxidant nutrients. Given that our dietary intake typically includes multiple antioxidants, the CDAI seems to exert a comprehensive effect on individuals' pro-antioxidant status and underscores the advantages of thorough assessments of antioxidant exposure [17].

Reference:

[17]. Wang M, Huang ZH, Zhu YH, He P, Fan QL. Association between the composite dietary antioxidant index and chronic kidney disease: evidence from NHANES 2011-2018. Food & function. 2023;14(20):9279-86. http://dx.doi.org/10.1039/d3fo01157g PMID: 37772927.

2. *Comment*: Based on that survey, what kind of food source that lead to increasing level of six nutrients by considering age, sex, race/ethnicity, education level, body mass index (BMI), poverty income ratio (PIR), Please write diet recommendation that is more effective and that is considered as a strategy in anemia prevention (add in your discuss).

2. Response: Based on your recommendations, we have checked the literature carefully and have revised the discussion to include specific dietary recommendations aimed at anemia prevention.

① (Manuscript, Discussion section, page 10, line 273-276): Andrea et al summarize the intricate interactions between antioxidant-rich foods and gut microbiota, inflammation, and obesity, positing that plant-based antioxidant foods, such as vegetables, fruits, and nuts, are essential for health maintenance [36].

② (Manuscript, Discussion section, page 10, line 281-285): Therefore, a balanced diet, such as the Mediterranean diet, along with specific foods like fish, fresh vegetables, and fruits, is recommended for individuals with low CDAI scores [36]. These foods are rich in essential components, including fiber, minerals, vitamins, and antioxidants, which can help prevent the occurrence of anemia.

Reference:

[36]. Deledda A, Annunziata G, Tenore GC, Palmas V, Manzin A, Velluzzi F. Diet-Derived Antioxidants and Their Role in Inflammation, Obesity and Gut Microbiota Modulation. Antioxidants (Basel, Switzerland). 2021;10(5). http://dx.doi.org/10.3390/antiox10050708 PMID: 33946864.

③ We have included effective dietary recommendations for the prevention of anemia in the conclusion section. Please refer to the conclusion section, page 12, line 325-331: This study revealed an inverse relationship between CDAI and the prevalence of

anemia, with more pronounced effect observed in male nonsmokers and nondiabetic individuals. These findings offer valuable insights for healthcare providers to develop more targeted anemia screening and prevention strategies. Furthermore, Our study suggest the potential role of dietary modifications, particularly for individuals with lower CDAI scores, including the adoption of antioxidant-rich dietary patterns, such as iron supplements and plant-based diets, to help reduce the prevalence of anemia.

Reviewer #2:

1. ***Comment***: How can the 24-hour dietary recall interview method explain the diversity of daily consumption, how to control consumption on holidays, during parties, when dieting or fasting?

1. Response: Thank you immensely for your inquiry, your insights are profoundly valuable.

1) In line with previous research, participants completed two such interviews. Please refer Manuscript, Method section, page 5, line 137-145: Data on dietary antioxidant intake were obtained from the average of two 24-hour dietary recall interviews conducted as part of the NHANES. The initial dietary recall was performed at mobile examination center (MEC) by trained interviewers who adhered to standardized protocol. During this face-to-face interview, detailed information regarding all food and beverages consumed by participants over the past 24 hours was collected. This was followed by second interview conducted via telephone 3 to 10 days later [23]. Utilizing average dietary intake data from two non-consecutive days is deemed more accurate than relying solely on data from a single day [24].

2) Given the diversity of daily consumption, it may not accurately reflect habitual eating patterns. Acknowledging these limitations, future research could benefit from utilizing multiple food recalls or food frequency questionnaires to obtain a more representative and comprehensive understanding of long-term dietary habits.

Reference:

previous research:

"Ni Y, et al. BMC Public Health. 2024; 24(1):2167. doi: 10.1186/s12889-024-19620-5."

"Zhu G, et al. Nutrients. 2022; 14(23):5026. doi: 10.3390/nu14235026"

"Wan H, et al. Sci Rep. 2024;14(1):2415. doi: 10.1038/s41598-024-52806-w."

[23]. Pannucci TE, Thompson FE, Bailey RL, Dodd KW, Potischman N, Kirkpatrick SI, et al. Comparing Reported Dietary Supplement Intakes between Two 24-Hour Recall Methods: The Automated Self-Administered 24-Hour Dietary Assessment Tool and the Interview-Administered Automated Multiple Pass Method. Journal of the Academy of Nutrition and Dietetics. 2018;118(6):1080-6. http://dx.doi.org/10.1016/j.jand.2018.02.013 PMID: 29803270.

[24]. Yang T, Yi J, He Y, Zhang J, Li X, Ke S, et al. Associations of Dietary Fats with All-Cause Mortality and Cardiovascular Disease Mortality among Patients with Cardiometabolic Disease. Nutrients. 2022;14(17). http://dx.doi.org/10.3390/nu14173608 PMID: 36079863.

2. **Comment**: How to overcome recall bias in this study?

2. Response: Thank you for your professional review. And we clarified that limitation in the discussion section.

We utilized 24-hour dietary recalls to evaluate dietary nutrient intake, relying on self-reported data that may be susceptible to recall bias. We acknowledge this limitation, which is explicitly addressed in our manuscript. (Manuscript, Discussion section, page 11, line 311-313)

3. **Comment**: How to control that the results of this analysis do not occur by chance, not because of the large number of samples? Are there other dominant factors that can interfere with the relationship between the two, besides the existing variables?

3. **Response**: Thank you for pointing this out. As you are concerned, several issues need to be addressed, which are replied to in detail as below.

1) This study employed weighted multiple logistic regression analysis to control for confounding variables, thereby enhancing the robustness of our findings.

2) Although this study accounted for numerous clinical variables, potential unmeasured confounders, such as medication use and laboratory indicators (including

liver and kidney function, iron levels, vitamins), may still influence CDAI levels and their correlation with outcomes. Consequently, future research should aim to explore more comprehensively the specific associations between these variables. (Manuscript, Discussion section, page 11, line 319-322)

4. **Comment**: How to improve the results of the relationship between the two, considering that the study design used was cross-sectional?

4. Response: Thanks for your significant reminding. We have included this limitation in the revised manuscript.

The cross-sectional design of this study inherently limits the ability to establish a causal relationship between the variables. Consequently, prospective multicenter studies will be necessary in the near future to validate our findings. (Manuscript, Discussion section, page 11, line 313-315)

Reviewer #3:

Dear Authors, I appreciate your efforts and hard work. I have a few recommendations.

Comment: Rephrase the opening sentence of the abstract: "Recent studies have revealed that..." This statement requires references. You may report it as there is a documented association between,,,, Add extra details to the method part.

Response: Thank you sincerely for dedicating your time to reviewing our manuscript and for your constructive feedback. We have rephrased the opening sentence of the abstract to state:

(Manuscript, Abstract section, page 2, line 35-36) : There is increasing acknowledgment of the potential role that diet rich in antioxidants may play in the prevention of anemia.

Background

Comment: Please elaborate on the relationship between anemia and antioxidants, and how antioxidants diminish anemia. Protein synthesis? What biological reaction affected by the oxidative stress? What physiological pathway is affect?

Response: We sincerely appreciate the valuable and constructive comments. We have expanded the introduction section to include detailed discussion on the relationship between anemia and antioxidants.

(Manuscript, Introduction section, page 3, line 87-91) Halima et al found that antioxidants exert protective effect against anemia and provide significant alternative benefits for RBC function. This is achieved by preventing lipid peroxidation in RBCs, increasing levels of reduced glutathione (GSH), and reducing RBC permeability [15].

Reference:

[15]. Al Balushi H, Hannemann A, Rees D, Brewin J, Gibson JS. The Effect of Antioxidants on the Properties of Red Blood Cells From Patients With Sickle Cell Anemia. Frontiers in physiology. 2019;10:976. http://dx.doi.org/10.3389/fphys.2019.00976 PMID: 31456691.

Methodology

Comment: i Have concern about the anemia cut score according to smoking status and living in high altitude areas? both affect the Hb cut score? Also you studies only iron deficiency anemia? not all types? clarify and justify why?

Response: Thanks for your significant reminding. We have checked out the relevant information and understand your comments.

1) According to national health industry standards, individuals who smoke or reside at altitudes exceeding 1,000 meters must make adjustments in accordance with the "WS/T 441-2013 Population Anemia Screening Method". Specifically, for every 1,000 meters of altitude increase in plateau regions, hemoglobin levels should increase by 4%. However, elevation data are not available in the NHANES database. It is important to note that in other comparable NHANES studies, the anemia data utilized were based on laboratory diagnoses, which we recognize as limitation of the current study. We have included this limitation in the revised manuscript. The details are as follows:

(Manuscript, Discussion section, page 11, line 316-318) Thirdly, the anemia data utilized in this study were derived from laboratory diagnoses and may not have accounted for the potential contributions of a history of anemia or biological

differences, such as smoking or an increased incidence of thalassemia.

Reference:

"Chen S, et al. Front Immunol. 2024;15:1391573. doi: 10.3389/fimmu.2024.1391573."

"Wang Y, et al. Ann Hematol. 2023;102(10):2651-2658. doi: 10.1007/s00277-023-05374-4."

"Drury KE, et al. JAMA Pediatr. 2016;170(1):29-34. doi: 10.1001/jamapediatrics.2015.3065."

2) This study utilized laboratory-diagnosed anemia data and examined all types of anemia, however, it did not assess laboratory data for iron abnormalities. Consequently, the potential contributions of iron deficiency or other causes of anemia to the incidence reported here remain undetermined, we recognize as limitation of the current study.

Comment: In your exclusion criteria, other than excluding the missing data, do you include chronic GI diseases, malabsorption disorders, and pregnancy?

Response: Thank you for your professional review. Due to the limited data on chronic gastrointestinal diseases and malabsorption disorders in the NHANES database, as well as references to other anemia-related literature, only pregnant individuals (N = 1,051) were excluded from our study.

Please refer to Manuscript, Methods section page 5, line 127-128: After excluding individuals without hemoglobin data (N = 14,320), those lacking CDAI data (N = 4,656), and pregnant participants (N = 1,051).

Reference:

1."Csaba P Kovesdy, et al. Clinical Kidney Journal, 2023; 16(2), 303:311. doi: 10.1093/ckj/sfac240."

2."Chen S, et al. Front Immunol. 2024; 15:1391573. doi: 10.3389/fimmu.2024.1391573"

The disucsison

Comment: It would be beneficial to include earlier research on the relationship between diet consumption and anemia (which are numerous). To support your hypothesis, compare your results with those of these studies.

Response: Thank you again for your thorough review and valuable comments. And

we have checked the earlier literature carefully and have made the corrections. The details as follows:

1) (Manuscript, Discussion section, page 10, line 269-273): In addition, studies focusing on specific anemic populations, such as patients with β- thalassemia, provide further insight into the relationship between anemia severity and antioxidant defenses. Allen et al suggest that the severity of anemia in these patients is linked to depletion of antioxidant defenses, indicating that antioxidant supplementation may be beneficial [35].

2) (Manuscript, Discussion section, page 10, line 277-279) : Meanwhile, Jacques et al investigated the impact of various nutrients, including antioxidants like vitamin A and vitamin C, on iron metabolism to mitigate the risk of anemia [37].

3) (Manuscript, Discussion section, page 10, line 279-285) : This finding aligns with our results, which show that higher CDAI scores are associated with lower prevalence of anemia, thereby emphasizing the potential therapeutic role of antioxidants. Therefore, a balanced diet, such as the Mediterranean diet, along with specific foods like fish, fresh vegetables, and fruits, is recommended for individuals with low CDAI scores [36]. These foods are rich in essential components, including fiber, minerals, vitamins, and antioxidants, which can help prevent the occurrence of anemia.

Reference:

[35]. Allen A, Perera S, Mettananda S, Rodrigo R, Perera L, Darshana T, et al. Oxidative status in the β-thalassemia syndromes in Sri Lanka; a cross-sectional survey. Free radical biology & medicine. 2021;166:337-47. http://dx.doi.org/10.1016/j.freeradbiomed.2021.02.028 PMID: 33677065.

[36]. Deledda A, Annunziata G, Tenore GC, Palmas V, Manzin A, Velluzzi F. Diet-Derived Antioxidants and Their Role in Inflammation, Obesity and Gut Microbiota Modulation. Antioxidants (Basel, Switzerland). 2021;10(5). http://dx.doi.org/10.3390/antiox10050708 PMID: 33946864.

[37]. Berger J, Wieringa FT, Lacroux A, Dijkhuizen MA. Strategies to prevent iron deficiency and improve reproductive health. Nutrition reviews. 2011;69 Suppl 1:S78-86. http://dx.doi.org/10.1111/j.1753-4887.2011.00436.x PMID: 22043887.

Yours sincerely,

Xiaoxia Gou, MD

Department of Head and Neck Oncology

The Second Affiliated Hospital of Zunyi Medical University

Zunyi, Guizhou 563000,China.

E-mail: gouxx2020@163.com
