# Supplementary material for: Association between composite dietary antioxidant indices and anemia: NHANES 2003–2018
Source: PLoS One. 2025 Jan 2;20(1):e0316397. doi: 10.1371/journal.pone.0316397 (PMC11694997; doi:10.1371/journal.pone.0316397)
Supplement: Attachment — Submitted filename: PONE-D-24-28259_reviewer.docx [file pone.0316397.s004.docx]

PLOS ONE

Association between composite dietary antioxidant indices and anemia: NHANES

2003–2018

--Manuscript Draft--

| Manuscript Number: | PONE-D-24-28259 |
| --- | --- |
| Article Type: | Research Article |
| Full Title: | Association between composite dietary antioxidant indices and anemia: NHANES 2003–2018 |
| Short Title: | CDAI and anemia |
| Corresponding Author: | Qian Wu  The Second Affiliated Hospital of Zunyi Medical University Zunyi, CHINA |
| Keywords: | Composite Dietary Antioxidant Index; Anemia; National Health and Nutrition Examination Survey |
| Abstract: | Abstract  Background: Recent studies have revealed that a diet high in antioxidants could play a role in preventing anemia. The composite dietary antioxidant index (CDAI) is used to measure antioxidant intake. Nevertheless, the exact link between CDAI and anemia remains uncertain.  Methods: Utilizing datas from the 2003-2018 National Health and Nutrition Examination Survey (NHANES) database, the CDAI is a comprehensive measure of the intake of six dietary antioxidants (vitamin A, vitamin C, vitamin E, zinc, selenium, carotene) obtained from 24-hour dietary recall interviews. Weighted multivariate logistic regression analysis to investigate the association between CDAI and anemia.  Furthermore, subgroup analyses were performed to enhance datas reliability.  Results: A total of 34,675 participants were included in the study. The unadjusted model showed negative association between CDAI and anemia (OR: 0.95; 95%CI: 0.94-0.96; P < 0.0001). After adjusting for all covariates , with each increase in CDAI level linked to 3% lower risk of anemia (OR: 0.97; 95%CI: 0.96-0.98; P < 0.0001). Moreover, when CDAI was categorized into quartiles, the observed trend persisted (P <0.0001). The restricted cubic spline (RCS) analysis revealed linear negative relationship between CDAI and anemia (P for nonlinearity = 0.619). Subgroup analysis of the antioxidant components of CDAI revealed that vitamins E, zinc, carotene, and selenium were independently associated with lower risk of anemia. Except for gender, smoking consumption, diabetes and hypertension, no statistically significant interactions were found in any subgroup analysis (P < 0.05 for interaction). Conclusion: An analysis of the NHANES database revealed a correlation between higher CDAI levels and a reduced risk of anemia. |
| Order of Authors: | Qian Wu |
|  | Zhiyu Wang |
|  | Jieyu Xia |
|  | Hui Xu |
|  | Gang Huang |
|  | guangyong Feng |
|  | Xiaoxia Gou |
| Additional Information: |  |
| Question | Response |
| Financial Disclosure  Enter a financial disclosure statement that describes the sources of funding for the | Yes |

| work included in this submission. Review the [submission guidelines](http://journals.plos.org/plosone/s/submission-guidelines#loc-financial-disclosure-statement) for detailed requirements. View published research articles from [PLOS ONE](https://journals.plos.org/plosone/) for specific examples.  This statement is required for submission and will appear in the published article if the submission is accepted. Please make sure it is accurate. |  |
| --- | --- |
| Funded studies Enter a statement with the following details: - Initials of the authors who received each award - Grant numbers awarded to each author - The full name of each funder - URL of each funder website - Did the sponsors or funders play any role in the study design, data collection and analysis, decision to publish, or preparation of the manuscript? |  |
| Did you receive funding for this work? |  |
| Please add funding details.   as follow-up to "Financial Disclosure  Enter a financial disclosure statement that describes the sources of funding for the work included in this submission. Review the [submission guidelines](http://journals.plos.org/plosone/s/submission-guidelines#loc-financial-disclosure-statement) for detailed requirements. View published research articles from [PLOS ONE](https://journals.plos.org/plosone/) for specific examples.  This statement is required for submission and will appear in the published article if the submission is accepted. Please make sure it is accurate. | 1.Guizhou Provincial Department of Science and Technology project  2.Science and Technology Program of Guizhou Anti-Cancer Association |

| Funded studies Enter a statement with the following details: - Initials of the authors who received each award - Grant numbers awarded to each author - The full name of each funder - URL of each funder website - Did the sponsors or funders play any role in the study design, data collection and analysis, decision to publish, or preparation of the manuscript? |  |
| --- | --- |
| Did you receive funding for this work?" |  |
| Please select the country of your main research funder (please select carefully as in some cases this is used in fee calculation).   as follow-up to "Financial Disclosure  Enter a financial disclosure statement that describes the sources of funding for the work included in this submission. Review the [submission guidelines](http://journals.plos.org/plosone/s/submission-guidelines#loc-financial-disclosure-statement) for detailed requirements. View published research articles from [PLOS ONE](https://journals.plos.org/plosone/) for specific examples.  This statement is required for submission and will appear in the published article if the submission is accepted. Please make sure it is accurate. | CHINA - CN |
| Funded studies Enter a statement with the following details: - Initials of the authors who received each award - Grant numbers awarded to each author - The full name of each funder - URL of each funder website - Did the sponsors or funders play any role in the study design, data collection and analysis, decision to publish, or preparation of the manuscript? |  |
| Did you receive funding for this work?" |  |
| Competing Interests | The authors have declared that no competing interests exist. |

| Use the instructions below to enter a competing interest statement for this submission. On behalf of all authors, disclose any [competing interests](http://journals.plos.org/plosone/s/competing-interests) that could be perceived to bias this work—acknowledging all financial support and any other relevant financial or nonfinancial competing interests.  This statement is required for submission and will appear in the published article if the submission is accepted. Please make sure it is accurate and that any funding sources listed in your Funding Information later in the submission form are also declared in your Financial Disclosure statement.  View published research articles from [PLOS ONE](http://journals.plos.org/plosone/) for specific examples. |  |
| --- | --- |
| NO authors have competing interests Enter: The authors have declared that no competing interests exist. Authors with competing interests Enter competing interest details beginning with this statement: I have read the journal's policy and the authors of this manuscript have the following competing interests: [insert competing interests here] |  |
| * typeset |  |
| Ethics Statement  Enter an ethics statement for this submission. This statement is required if the study involved:   - Human participants - Human specimens or tissue - Vertebrate animals or cephalopods - Vertebrate embryos or tissues | NA |

| • Field research  Write "N/A" if the submission does not require an ethics statement.  General guidance is provided below. Consult the [submission guidelines](http://journals.plos.org/plosone/s/submission-guidelines#loc-guidelines-for-specific-study-types) for detailed instructions. Make sure that all information entered here is included in the Methods section of the manuscript. |  |
| --- | --- |

Format for specific study types

Human Subject Research (involving human

participants and/or tissue)

Give the name of the institutional review

board or ethics committee that approved the

study

•

Include the approval number and/or a

statement indicating approval of this

research

•

Indicate the form of consent obtained

(

written/oral) or the reason that consent was

not obtained (e.g. the data were analyzed

anonymously)

•

Animal Research (involving vertebrate

animals, embryos or tissues)

Provide the name of the Institutional Animal

Care and Use Committee (IACUC) or other

relevant ethics board that reviewed the

study protocol, and indicate whether they

approved this research or granted a formal

waiver of ethical approval

•

Include an approval number if one was

obtained

•

If the study involved

non-human primates

,

add

additional details

about animal welfare

and steps taken to ameliorate suffering

•

If anesthesia, euthanasia, or any kind of

animal sacrifice is part of the study, include

briefly which substances and/or methods

were applied

•

Field Research

Include the following details if this study

involves the collection of plant, animal, or

other materials from a natural setting:

Field permit number

•

Name of the institution or relevant body that

granted permission

•

Data Availability

Authors are required to make all data

underlying the findings described fully

available, without restriction, and from the

time of publication. PLOS allows rare

exceptions to address legal and ethical

concerns. See the

[PLOS Data Polic](http://journals.plos.org/plosone/s/data-availability)

[y](http://journals.plos.org/plosone/s/data-availability)

and

[FA](http://journals.plos.org/plosone/s/data-availability#loc-faqs-for-data-policy)

[Q](http://journals.plos.org/plosone/s/data-availability#loc-faqs-for-data-policy)

for detailed information.

Yes - all data are fully available without restriction

| A Data Availability Statement describing where the data can be found is required at submission. Your answers to this question constitute the Data Availability Statement and will be published in the article, if accepted. |  |
| --- | --- |
| Important: Stating ‘data available on request from the author’ is not sufficient. If your data are only available upon request, select ‘No’ for the first question and explain your exceptional situation in the text box. |  |
| Do the authors confirm that all data underlying the findings described in their manuscript are fully available without restriction? |  |
| Describe where the data may be found in full sentences. If you are copying our sample text, replace any instances of XXX with the appropriate details.   - If the data are held or will be held in a public repository, include URLs, accession numbers or DOIs. If this information will only be available after acceptance, indicate this by ticking the box below. For example: All XXX files   are available from the XXX database (accession number(s) XXX, XXX.).   - If the data are all contained within the manuscript and/or Supporting Information files, enter the following: All relevant data are within the manuscript and its Supporting Information files. - If neither of these applies but you are able to provide details of access elsewhere, with or without limitations, please do so. For example:   Data cannot be shared publicly because of [XXX]. Data are available from the XXX Institutional Data Access / Ethics Committee (contact via XXX) for researchers who meet the criteria for access to confidential data.  The data underlying the results presented in the study are available from (include the name of the third party | All relevant data are within the manuscript and its Supporting information files. |

| and contact information or URL).  • This text is appropriate if the data are owned by a third party and authors do not have permission to share the data.  * typeset |  |
| --- | --- |
| Additional data availability information: |  |

Manuscript	[Click here to access/download;Manuscript;manuscript.docx](https://www2.cloud.editorialmanager.com/pone/download.aspx?id=36889515&guid=75f3c782-db23-4ca4-b38d-96ab19aef684&scheme=1)

# Association between composite dietary antioxidant indices and anemia: NHANES 2003–2018

## Abstract

**Background**: Recent studies have revealed that a diet high in antioxidants could play a role in preventing anemia. The composite dietary antioxidant index (CDAI) is used to measure antioxidant intake. Nevertheless, the exact link between CDAI and anemia remains uncertain.

**Methods**: Utilizing datas from the 2003-2018 National Health and Nutrition Examination Survey (NHANES) database, the CDAI is a comprehensive measure of the intake of six dietary antioxidants (vitamin A, vitamin C, vitamin E, zinc, selenium, carotene) obtained from 24-hour dietary recall interviews. Weighted multivariate logistic regression analysis to investigate the association between CDAI and anemia. Furthermore, subgroup analyses were performed to enhance datas reliability.

**Results:** A total of 34,675 participants were included in the study. The unadjusted model showed negative association between CDAI and anemia (OR: 0.95; 95%CI: 0.94-0.96; *P* < 0.0001). After adjusting for all covariates , with each increase in CDAI level linked to 3% lower risk of anemia (OR: 0.97; 95%CI: 0.96-0.98; *P* < 0.0001). Moreover, when CDAI was categorized into quartiles, the observed trend persisted (*P* <0.0001). The restricted cubic spline (RCS) analysis revealed linear negative relationship between CDAI and anemia (*P* for nonlinearity = 0.619). Subgroup analysis of the antioxidant components of CDAI revealed that vitamins E, zinc, carotene, and selenium were independently associated with lower risk of anemia. Except for gender, smoking consumption, diabetes and hypertension, no statistically significant interactions were found in any subgroup analysis (*P* < 0.05 for interaction).

**Conclusion:** An analysis of the NHANES database revealed a correlation between higher CDAI levels and a reduced risk of anemia.

**Keywords**: Composite Dietary Antioxidant Index; Anemia; National Health and Nutrition Examination Survey.

# Introduction

Anemia is a widespread global health concern that has been consistently linked to various negative outcomes in recent years [1]. It is characterized by an imbalance between the production and destruction of red blood cells (RBCs), resulting in inadequate oxygen delivery to vital tissues like the brain and heart [2]. The diagnosis of anemia typically relies on the hemoglobin level in the blood [3]. This condition can have detrimental effects on cognitive and physical functions [4], leading to decreased economic productivity and higher morbidity and mortality rates [5], presenting a significant health challenge in modern society.

Oxidative stress is defined as an imbalance between the production of reactive oxygen species (ROS) and antioxidant defense mechanisms [6], and is acknowledged as a major factor in various conditions like inflammation, aging, cancer, and cardiovascular diseases. The harmful effects of oxidative stress include the oxidation of cellular components, which can lead to cell death and organ damage through mechanisms [7] such as DNA damage, lipid peroxidation [8], and protein oxidation [9]. This damage further stimulates the production of ROS, worsening the underlying disease [10]. Numerous studies have emphasized a significant link between oxidative stress and the onset of anemia [11-13]. A modifiable risk factor for reducing oxidative stress is diet [14]. By including dietary antioxidants, individuals can alleviate or prevent oxidative stress-related diseases by neutralizing the harmful effects of ROS [15]. Consistent consumption of antioxidants can reduce oxidative stress levels and enhance the body’s ability to withstand it [16]. Recent research has highlighted the importance of consuming an antioxidant-rich diet to prevent anemia. The CDAI was developed by Wright et al as a tool to assess antioxidant intake. This index considers key nutrients such as vitamins A, vitamins C, and vitamins E, zinc, selenium, and carotenoids. While previous studies have mainly examined the relationship between CDAI and different diseases like cancer [17], coronary heart disease [=18], chronic kidney disease [19], and diabetes [20], showing a positive link between higher CDAI scores and reduced prevalence of these diseases, however, the specific connection between CDAI and anemia is yet to be conclusively determined.

A cross-sectional study was conducted using data from the National Health and Nutrition Examination Survey (NHANES) to assess the relationship between CDAI and anemia. The hypothesis posited that higher CDAI scores would correlate with a decreased prevalence of anemia.

# Materials and methods

## NHANES database

The analysis is based on data from the NHANES, a cross-sectional survey conducted by the Centers for Disease Control and Prevention and the National Center for Health Statistics. The survey’s objective is to evaluate the health and nutrition status of a representative population, encompassing institutionalized individuals^21^. Since 1999, NHANES has been an ongoing study that releases data every two years^22^. The database is available for free download from the official website. Approval for the study was obtained from the National Center for Health Statistics Research Ethics Review Board, and all participants provided written informed consent, and no external ethic approval was required for this study.

## Study population

This study utilized NHANES data from 2003 to 2018, involving a total of 80,312 participants. After excluding 14,320 individuals without hemoglobin data and 4,656 without CDAI data, further removal of cases with missing covariate data resulted in a final analysis sample of 34,439 participants. The exclusion criteria flow chart can be observed in Fig. 1**.**

## Exposure and Outcomes

According to World Health Organization (WHO) guidelines, patients with anemia are defined as having hemoglobin (HB) levels below 12 g/dL for women and below 13 g/dL for men [23].

The CDAI was developed using data from NHANES 24-hour dietary recall interviews conducted. The index utilized dietary data from two separate interviews to provide a more accurate representation of dietary intake [24]. To evaluate the overall exposure to dietary antioxidants, the intake of six key antioxidants (vitamin A, vitamin C, vitamin E, zinc, selenium, and carotenoids) was analyzed and quantified using to calculate the CDAI [25]. This involved standardizing the intake levels of the six antioxidants by subtracting the mean and dividing by the standard deviation, followed by summing up the standardized values:

## Covariates

Potential confounders considered in this study including age, sex, race/ethnicity, education level, body mass index (BMI), poverty income ratio (PIR), smoking consumption, alcohol consumption, as well as comorbidities like hypertension, diabetes, cancer, and hyperlipidemia. Age was categorized into 40 years, ethnicity into non-Hispanic White, non-Hispanic Black, Mexican American, and others, and education levels into less than high school, high school, and more than high school.

Marital status was divided into married/living with partner or widowed/divorced/separated/never married. PIR was categorized as low (≤2.14) and high (≥2.14). BMI was categorized as thin/normal (<=18.5kg/m2, 18.6-24.9kg/m2), overweight (25.0-29.9 kg/m2), and obese (>=30 kg/m2).

Smoking status was categorized into two groups: smokers, defined as those who had smoked at least 100 cigarettes in their lifetime (coded as ‘yes’), and non-smokers (coded as ‘no’) [26]. Alcohol consumption was classified as ‘yes’ for those who had consumed at least 12 alcoholic drinks in the past year, and ‘no’ for others [27]. Participants were considered to have a history of diabetes, hypertension, and hyperlipidemia if they reported physicians diagnosis of these conditions. The diagnosis of cancer required meeting two criteria: (1) a positive response to ‘ever been told they have cancer or a malignancy of any kind’ (variable mcq220); (2) providing details for the inquiry ‘what kind of cancer?’ (variable mcq230A).

## Statistical analysis

Participants were categorized into four groups based on quartiles CDAI. Continuous variables were presented as mean±standard deviation (mean±SD), while categorical variables were expressed as percentages. The associations between baseline characteristics and CDAI quartiles were evaluated using chi-square or t tests. Weighted logistic regression model was employed to investigate the relationship between CDAI and anemia, with results reported as adjusted odds ratio (OR) and 95% confidence intervals (CI). Model 1 had no adjustments, while model 2 included adjustments for gender, age, education level, and race. Model 3 further adjusted for PIR, BMI, smoking, drinking, hypertension, diabetes, and hyperlipidemia as covariates in addition to those in Model 2. CDAI was analyzed both as continuous and categorical variable to explore correlations. All statistical analyses were conducted using R 4.3.3 with appropriate weights, and statistical significance was defined as *P* < 0.05.

## Ethics statement

The survey procedures are in accordance with the standards outlined in the declaration of Helsinki [28]. All information from the NHANES program is freely available to the public, therefore, the approval of the medical ethics committee board was not required [29].

# Results

## Baseline characteristics

This study involved 34,675 patients who met strict inclusion and exclusion criteria, with 3,885 (7.19%) of them diagnosed anemia. The average age of the participants was 47.12±0.22 years. Patients were categorized into 4 groups based on CDAI quartiles, and their baseline characteristics are presented in table 1. Significant differences in age, gender, race, education level, smoking consumption, alcohol consumption, PIR, BMI, diabetes, cancer, hypertension and hyperlipidemia were observed (*P* < 0.001). Higher GDAI levels were associated with certain characteristics such as younger age, white female, non-drinkers, non-smokers, married status, higher BMI, higher PIR, higher education level, absence of diabetes, cancer, hypertension, and anemia, but presence of hyperlipidemia. Univariate and multivariate logistic analyses showed significant link between CDAI and anemia (*P* <0.001) (Supplementary Table 1).

## The association between CDAI and anemia

Table 2 presents the results of the weighted logistic regression analysis investigating the relationship between anemia and CDAI. The analysis demonstrated robust negative correlation between CDAI and anemia when considering CDAI as continuous variable in model 1 (OR: 0.95; 95%CI: 0.94-0.96), model 2 (OR:0.97; 95%CI: 0.95-0.98) and model 3 (OR:0.97; 95%CI: 0.96-0.98). Particularly noteworthy is the significant trend in model 3 (*P* for trend <0.0001) when CDAI was categorized into quartiles, indicating that higher CDAI scores were linked to lower likelihood of anemia. To further confirm this association, restricted cubic spline (RCS) analysis with 3 knots was performed, revealing linear negative correlation between CDAI and anemia (*P* for nonlinearity = 0.619), as depicted in Fig. 2.

## The association between antioxidant components and anemia

The relationship between the six antioxidant components of CDAI and anemia was further examined (Table 3), it was observed that vitamin A was linked to anemia in model 1 (OR:1.00; 95%CI:1.00-1.00, *P* = 0.01). Vitamin C showed an association with anemia in models 1 and 2 (OR:1.00; 95%CI:1.00-1.00, *P* = 0.03 or *P* =0.02). After adjusting for all variables, zinc (OR: 0.98; 95% CI:0.97-0.99, *P* < 0.0001), vitamin E (OR:0.98; 95%CI:0.98-0.99, *P* <0.001), carotene (OR:1.00; 95% CI:1.00-1.00, *P* < 0.0001), and selenium (OR: 1.00; 95% CI:1.00-1.00, *P* = 0.01) were identified as independent factors associated with anemia.

## Subgroup analysis

To further investigate the influence of CDAI on anemia risk within specific subgroups, stratified analysis was performed based on gender, age, race, PIR, BMI, hypertension, diabetes, and hyperlipidemia. Results indicated that except for gender, smoking consumption, diabetes and hypertension, there were no significant interactions between CDAI and anemia risk (*P* for interaction > 0.05, Fig. 3).

# Discussion

This study is the first to investigate the relationship between CDAI and the risk of anemia based on data from NHANES. Upon adjusting for potential confounders, a negative association between CDAI and anemiain American adults was observed, with a linear trend established through dose-response analysis. Additionally, subgroup analysis indicated no significant interactions among various subgroups, except for hypertension, smoking consumption, and gender.

There is growing evidence indicating that oxidative stress plays a significant role in the development and advancement of anemia. RBCs are particularly susceptible to oxidative damage when exposed to ROS from various sources [30]. Enika et al utilized mouse model, demonstrating that anemia can elevate oxidative stress in RBCs [31]. Similarly, Yoshihito et al established a connection between oxidative stress and autoimmune hemolytic anemia in mice, highlighting a correlation between the severity of anemia and intracellular ROS levels [32]. A recent study revealed that individuals with sickle cell anemia (SCA) experience disruptions in cellular redox balance and purine metabolite processing due to oxidative stress and changes in antioxidant markers [33].

Diet plays a crucial role in managing the body’s oxidative stress levels [34], with dietary antioxidants being essential in lowering the risk of aging, cancer, diabetes, inflammation, liver disease, and cardiovascular disease. The fast-paced lifestyle of modern society leads to an increase in free radicals in the body, which can demage cells, tissues, and organs, ultimately impacting longevity. Antioxidants are key in fighting free radicals, thereby contributing to prevent both short-term and long-term diseases [35]. While research on the relationship between CDAI and anemia is currently limited, studies in other areas have gained significant attention. A study within NHANES demonstrated a negative linear correlation between CDAI and hypertension [36]. These results also propose that increasing CDAI levels through a diet rich in antioxidant nutrients could potentially reduce the occurrence of metabolic syndrome [14]. Our study reinforces these findings by showing an inverse relationship between CDAI levels and anemia in American adults. Specifically, higher CDAI scores were linked to a lower prevalence of anemia, suggesting the need for further investigation to solidify the connection between antioxidant intake and anemia.

Subsequently, this study explored the correlation between antioxidant components and anemia, discovering that vitamins E, zinc, carotene, and selenium were independently linked to anemia after adjusting for all potential confounders. Vitamin E is recognized as a crucial lipophilic antioxidant in biological membranes, capable of scavenging free radicals and acting as a chain-breaking antioxidant [37]. Severe vitamin E deficiency can lead to impaired immune response and hemolytic anemia caused by free radicals [38]. Sue et al highlighted that the lack of multiple trace element biomarkers (iron, zinc, selenium) was positively associated with anemia [39]. While Lisa et al showed that low zinc is an independent risk factor for anemia in school-age children and mediates the effect of low selenium on hemoglobin levels [40].

This study has several strengths. Firstly, it is the first cross-sectional survey to explore the relationship between CDAI and anemia, emphasizing the potential impact of dietary antioxidants in reducing anemia prevalence. Secondly, the findings indicate that the consumption of antioxidant- rich foods could potentially decrease the risk of anemia and offer dietary guidance to boost antioxidant intake for individuals with anemia. However, the study also presents limitations. Firstly, the dietary data in NHANES is based on self-reporting, which may introduce recall bias, and does not cover all dietary antioxidants. Secondly, the cross-sectional design makes it difficult to establish causal relationship between CDAI and anemia. Lastly, as the study was confined to American population, further research is necessary to confirm the findings in other countries.

## Conclusion

In summary, A cross-sectional study utilizing data from 8 periods (2003-2018) of the NHANES database revealed negative association between CDAI and anemia in the American adults, even after accounting for potential confounders. This study offers a novel approach to explore dietary intervention strategies for reducing the prevalence of anemia. Further large-scale prospective studies are necessary to comfirm these findings and offer more precise and efficient dietary recommendations for apreventing anemia.

## Reference

1. Ugo Lucca, et al. Anemia and the risk of dementia in the oldest-old: The Monzino 80-plus population-based study. *Alzheimer's & Dementia*. **16**, e044445 (2020).
2. Kassebaum, N. J. & GBD 2013 Anemia Collaborators. The Global Burden of Anemia. *Hematol Oncol Clin North Am* **30**, 247–308 (2016).
3. Bansal PG, Toteja GS, Bhatia N, Gupta S, Kaur M, Adhikari T, Garg AK. Comparison of haemoglobin estimates using direct & indirect cyanmethaemoglobin methods. *Indian J Med Res* 144(**4**), 566-571 (2016).
4. Kinyoki D, et al. Anemia prevalence in women of reproductive age in low- and middle-income countries between 2000 and 2018. *Nat Med* 27(**10**), 1761-1782 (2021).
5. Martinsson, A. et al. Anemia in the general population: prevalence, clinical correlates and prognostic impact. *Eur J Epidemiol.* **29,** 489–498 (2014).
6. Du W, Adam Z, Rani R, Zhang X, Pang Q. Oxidative stress in Fanconi anemia hematopoiesis and disease progression. *Antioxid Redox Signal* 10(**11**), 1909-21 (2008).
7. Fibach E, Dana M. Oxidative Stress in β-Thalassemia. *Mol Diagn Ther*. 23(**2**), 245-261 (2019).
8. Niki E. Antioxidants in relation to lipid peroxidation. *Chem Phys Lipids*. 44(**2-4**), 227-53(1987).
9. Abeyrathne EDNS, Nam K, Huang X, Ahn DU. Plant- and Animal-Based Antioxidants' Structure, Efficacy, Mechanisms, and Applications: A Review. Antioxidants (Basel).

11(**5**), 1025 (2022).

10. Aboderin FI, Oduola T, Davison GM, Oguntibeju OO. A Review of the Relationship between the Immune Response, Inflammation, Oxidative Stress, and the Pathogenesis of Sickle Cell Anaemia. Biomedicines. 11(**9**), 2413 (2023).
11. Meroño T, et al. Oxidative stress, HDL functionality and effects of intravenous iron administration in women with iron deficiency anemia. *Clin Nutr*. 36(**2**), 552-558 (2017).
12. Castilhos LG, et al. Increased oxidative stress alters nucleosides metabolite levels in sickle cell anemia. *Redox Rep*. 22(**6**), 451-459 (2017).
13. Podkowińska A, Formanowicz D. Chronic Kidney Disease as Oxidative Stress- and

Inflammatory-Mediated Cardiovascular Disease. Antioxidants (Basel). 9(**8**), 752 (2020).

14. Yingjie Zhu, et al. The association between composite dietary antioxidant index and the metabolic syndrome: NHANES 2007–2018. *Journal of Functional Foods*. **116**, 1756-4646 (2024).
15. Corrochano AR, et al. Invited review: Whey proteins as antioxidants and promoters of cellular antioxidant pathways. *J Dairy Sci*. 101(**6**), 4747-4761 (2018).
16. Ogura Y, Kitada M, Koya D. Sirtuins and Renal Oxidative Stress. *Antioxidants (Basel)*.

10(**8**), 1198 (2021).

17. Y. C. Yu , et al. Composite dietary antioxidant index and the risk of colorectal cancer: Findings from the Singapore Chinese Health Study, *Int. J. Cancer*. **150**, 1599 -1608.
18. Ma R, et al. Association between composite dietary antioxidant index and coronary heart disease among US adults: a cross-sectional analysis. *BMC Public Health*. 23(**1**), 2426 (2023).
19. Wang M, et al. Association between the composite dietary antioxidant index and chronic kidney disease: evidence from NHANES 2011-2018. *Food Funct.* 14(**20**), 9279-9286 (2023).
20. W. Wang , et al , Dietary Antioxidant Indices in Relation to All-Cause and Cause-Specific Mortality Among Adults With Diabetes: A Prospective Cohort Study, *Front. Nutr.* **9**, 849727 (2022).
21. Qing, L., Zhu, Y., Yu, C. et al. Exploring the association between dietary Inflammatory Index and chronic pain in US adults using NHANES 1999–2004. *Sci Rep*. **14**, 8726 (2024).
22. Scinicariello F, Buser MC, Feroe AG, Attanasio R. Antimony and sleep-related disorders: NHANES 2005-2008. *Environ Res*. **156**, 247-252 (2017).
23. Safiri S, Kolahi AA, Noori M, et al. Burden of anemia and its underlying causes in 204 countries and territories, 1990-2019: results from the Global Burden of Disease Study 2019. *J Hematol Oncol*. 14(**1**), 185 (2021).
24. Yang T, Yi J, He Y, et al. Associations of Dietary Fats with All-Cause Mortality and Cardiovascular Disease Mortality among Patients with Cardiometabolic Disease. *Nutrients*. 14(**17**), 3608 (2022).
25. Xu, Q., Qian, X., Sun, F., et al. Independent and joint associations of dietary antioxidant intake with risk of post-stroke depression and all- cause mortality. *Journal of Affective Disorders*. **322**, 84–90 (2023).
26. Wang M, Li J, Peng H, et al. Patients with different types of arthritis may be at risk for major depression: results from the national health and nutrition examination survey 2007–2018. *Ann Palliat Med*. **10**, 5280–8 (2021).
27. King D, Xiang J. The dietary inflammatory index is associated with diabetes severity. *J Am Board Fam Med*. **32**, 801–6 (2019).
28. Xu H, Wen Q, et al. High Dietary Inflammatory Index increases the risk of female infertility: An analysis of NHANES 2013-2018. *Nutr Res*.**125**, 50-60 (2024).
29. Luo, J., Chen, T., Chen, Y. et al. The association between homocysteine and bacterial vaginosis: results from NHANES 2001–2004. *Sci Rep* **13**, 21388 (2023).
30. Sharma P, Puri N. A new role for mast cells as scavengers for clearance of erythrocytes damaged due to oxidative stress. *Immunol Lett*. **199**, 23-35 (2018).
31. Nagababu E, Gulyani S, Earley CJ, et al. Iron-deficiency anaemia enhances red blood cell oxidative stress. Free Radic Res. 42(**9**), 824-9 (2008).
32. Iuchi Y, Kibe N, Tsunoda S, et al. Implication of oxidative stress as a cause of autoimmune hemolytic anemia in NZB mice. Free Radic Biol Med. 48(**7**), 935-44 (2010).
33. Castilhos LG, de Oliveira JS, Adefegha SA, et al. Increased oxidative stress alters nucleosides metabolite levels in sickle cell anemia. *Redox Rep*. 22(**6**), 451-459 (2017).
34. Teng TQ, Liu J, Hu FF, et al. Association of composite dietary antioxidant index with prevalence of stroke: insights from NHANES 1999-2018. *Front Immunol*. **15**, 1306059 (2024).
35. Neha K, Haider MR, Pathak A, et al. Medicinal prospects of antioxidants: A review. *Eur J Med Chem*. **178**, 687-704 (2019).
36. Wu M, Si J, Liu Y, Kang L, Xu B. Association between composite dietary antioxidant index and hypertension: insights from NHANES. *Clin Exp Hypertens*. 45(**1**), 2233712 (2023).
37. TAKAHASHI, M., TSUCHIYA, J. and NIKI, E. Mode of Action of Vitamin E as Antioxidant in the Membranes as Studied by Spin Labeling. *Annals of the New York Academy of Sciences.* **570**, 521-523 (1989).
38. Jilani T, Iqbal MP. Vitamin E deficiency in South Asian population and the therapeutic use of alpha-tocopherol (Vitamin E) for correction of anemia. *Pak J Med Sci*. 34(**6**), 1571-1575 (2018).
39. MacDonell SO, Miller JC, Harper MJ, et al. Multiple Micronutrients, Including Zinc, Selenium and Iron, Are Positively Associated with Anemia in New Zealand Aged Care Residents. *Nutrients*. 13(**4**), 1072 (2021).
40. Houghton LA, Parnell WR, Thomson CD, et al. Serum Zinc Is a Major Predictor of Anemia and Mediates the Effect of Selenium on Hemoglobin in School-Aged Children in a Nationally Representative Survey in New Zealand. *J Nutr*. 146(**9**), 1670-6 (2016).

[Click here to access/download;Figure;Figure 1.tif](https://www2.cloud.editorialmanager.com/pone/download.aspx?id=36863234&guid=14b31a33-6a8d-4c73-8d89-5a4dc29dd327&scheme=1)

[Click here to access/download;Figure;Figure 2.tiff](https://www2.cloud.editorialmanager.com/pone/download.aspx?id=36863235&guid=4554c61a-9e6f-4605-8bb5-10dd27e459b0&scheme=1)

[Click here to access/download;Figure;Figure 3.tif](https://www2.cloud.editorialmanager.com/pone/download.aspx?id=36863236&guid=aadd2ef1-d62f-4d36-be91-e3903efb59cb&scheme=1)

Grapgical abstract

[Click here to access/download](https://www2.cloud.editorialmanager.com/pone/download.aspx?id=36863238&guid=b4f25b0d-e1f0-41af-a730-4e2573c40503&scheme=1)

[Supporting Information](https://www2.cloud.editorialmanager.com/pone/download.aspx?id=36863238&guid=b4f25b0d-e1f0-41af-a730-4e2573c40503&scheme=1)

[Graphical Absrtact.pdf](https://www2.cloud.editorialmanager.com/pone/download.aspx?id=36863238&guid=b4f25b0d-e1f0-41af-a730-4e2573c40503&scheme=1)

[Click here to access/download](https://www2.cloud.editorialmanager.com/pone/download.aspx?id=36863239&guid=76f6673a-d8ce-4e3f-a928-efe10c9dbf1c&scheme=1)

[Supporting Information Table 1.pdf](https://www2.cloud.editorialmanager.com/pone/download.aspx?id=36863239&guid=76f6673a-d8ce-4e3f-a928-efe10c9dbf1c&scheme=1)

[Click here to access/download](https://www2.cloud.editorialmanager.com/pone/download.aspx?id=36863240&guid=522f2212-54ab-428c-82c5-4682dc7e88a3&scheme=1)

[Supporting Information Table 2.pdf](https://www2.cloud.editorialmanager.com/pone/download.aspx?id=36863240&guid=522f2212-54ab-428c-82c5-4682dc7e88a3&scheme=1)

[Click here to access/download](https://www2.cloud.editorialmanager.com/pone/download.aspx?id=36863242&guid=2379f59e-f795-4280-b95b-4eb17df51abb&scheme=1)

[Supporting Information Table 3.pdf](https://www2.cloud.editorialmanager.com/pone/download.aspx?id=36863242&guid=2379f59e-f795-4280-b95b-4eb17df51abb&scheme=1)

Supplementary Table 1

[Click here to access/download Supporting Information](https://www2.cloud.editorialmanager.com/pone/download.aspx?id=36863244&guid=4adf76c0-2025-4015-b22f-d91069c68db7&scheme=1)

[Supplementary Table 1.pdf](https://www2.cloud.editorialmanager.com/pone/download.aspx?id=36863244&guid=4adf76c0-2025-4015-b22f-d91069c68db7&scheme=1)

code

[Click here to access/downloa](https://www2.cloud.editorialmanager.com/pone/download.aspx?id=36863265&guid=ca1e8f44-3eb4-4c67-a9ee-1e813d1ee4b0&scheme=1)

[d](https://www2.cloud.editorialmanager.com/pone/download.aspx?id=36863265&guid=ca1e8f44-3eb4-4c67-a9ee-1e813d1ee4b0&scheme=1)

[Supporting Informatio](https://www2.cloud.editorialmanager.com/pone/download.aspx?id=36863265&guid=ca1e8f44-3eb4-4c67-a9ee-1e813d1ee4b0&scheme=1)

[n](https://www2.cloud.editorialmanager.com/pone/download.aspx?id=36863265&guid=ca1e8f44-3eb4-4c67-a9ee-1e813d1ee4b0&scheme=1)

[code.tx](https://www2.cloud.editorialmanager.com/pone/download.aspx?id=36863265&guid=ca1e8f44-3eb4-4c67-a9ee-1e813d1ee4b0&scheme=1)

[t](https://www2.cloud.editorialmanager.com/pone/download.aspx?id=36863265&guid=ca1e8f44-3eb4-4c67-a9ee-1e813d1ee4b0&scheme=1)
